# Supplementary figures and images for: miRNA-target complementarity in cnidarians resembles its counterpart in plants
Source: EMBO Rep. 2025 Jan 2;26(3):836–59. doi: 10.1038/s44319-024-00350-z (PMC11811051; doi:10.1038/s44319-024-00350-z)

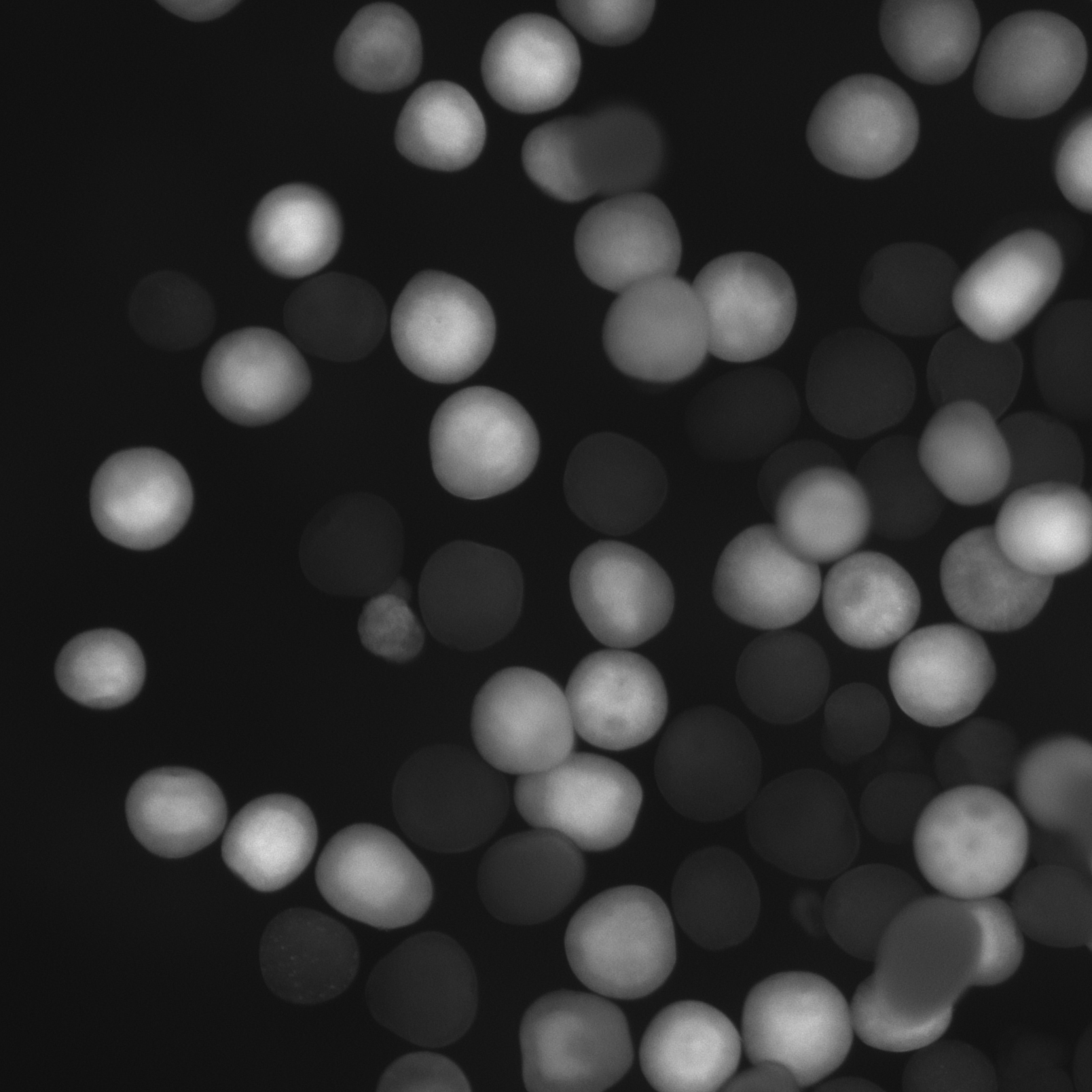

Supplement: Supplementary file 2 — Source data [file 44319_2024_350_MOESM2_ESM.zip › source data/Figure 1/1B/Nematostella transgene + mimiR seed.tif]

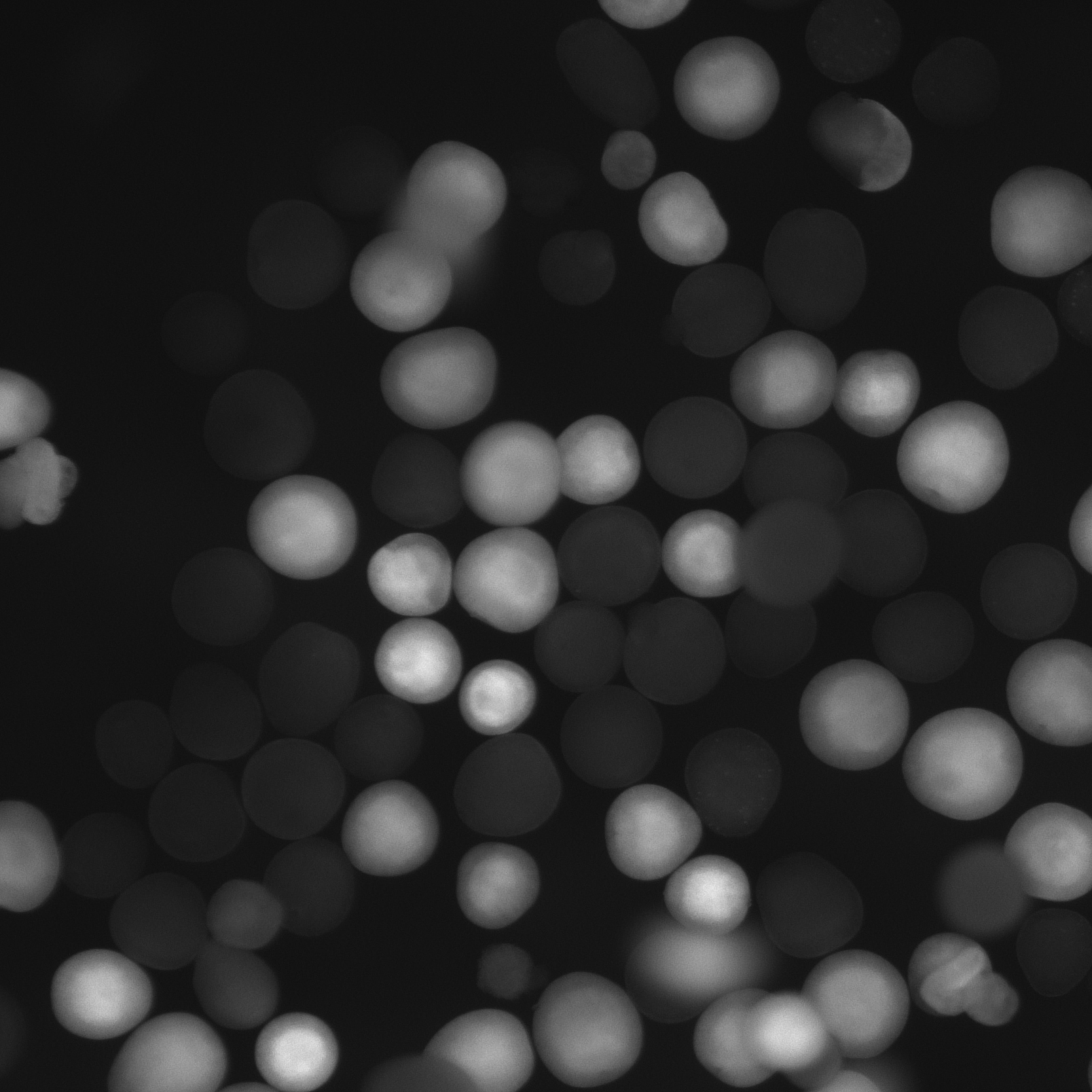

Supplement: Supplementary file 2 — Source data [file 44319_2024_350_MOESM2_ESM.zip › source data/Figure 1/1B/Nematostella transgene + negative control shRNA.tif]

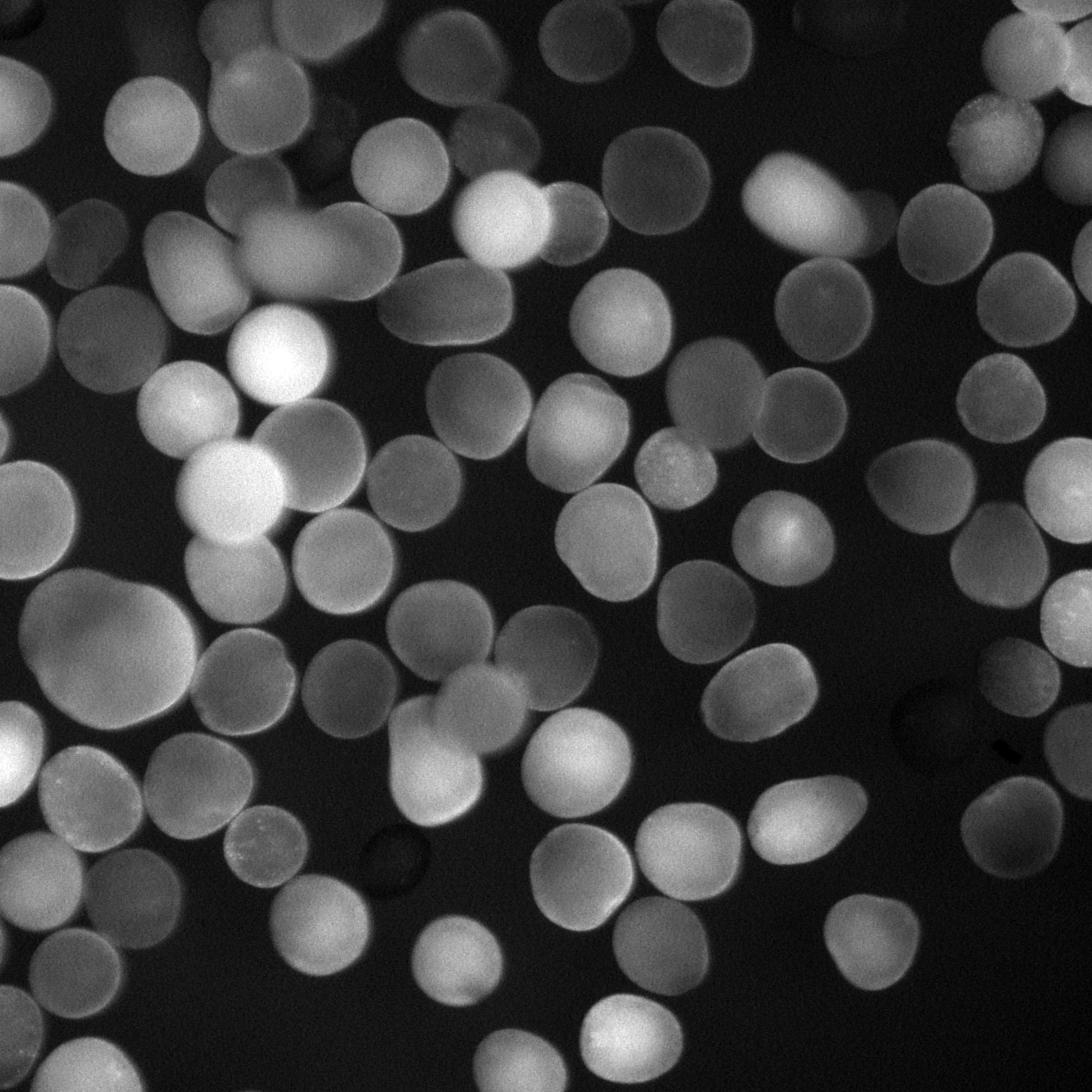

Supplement: Supplementary file 2 — Source data [file 44319_2024_350_MOESM2_ESM.zip › source data/Figure 1/1B/Nematostella transgene + positive control mimiR.tif]

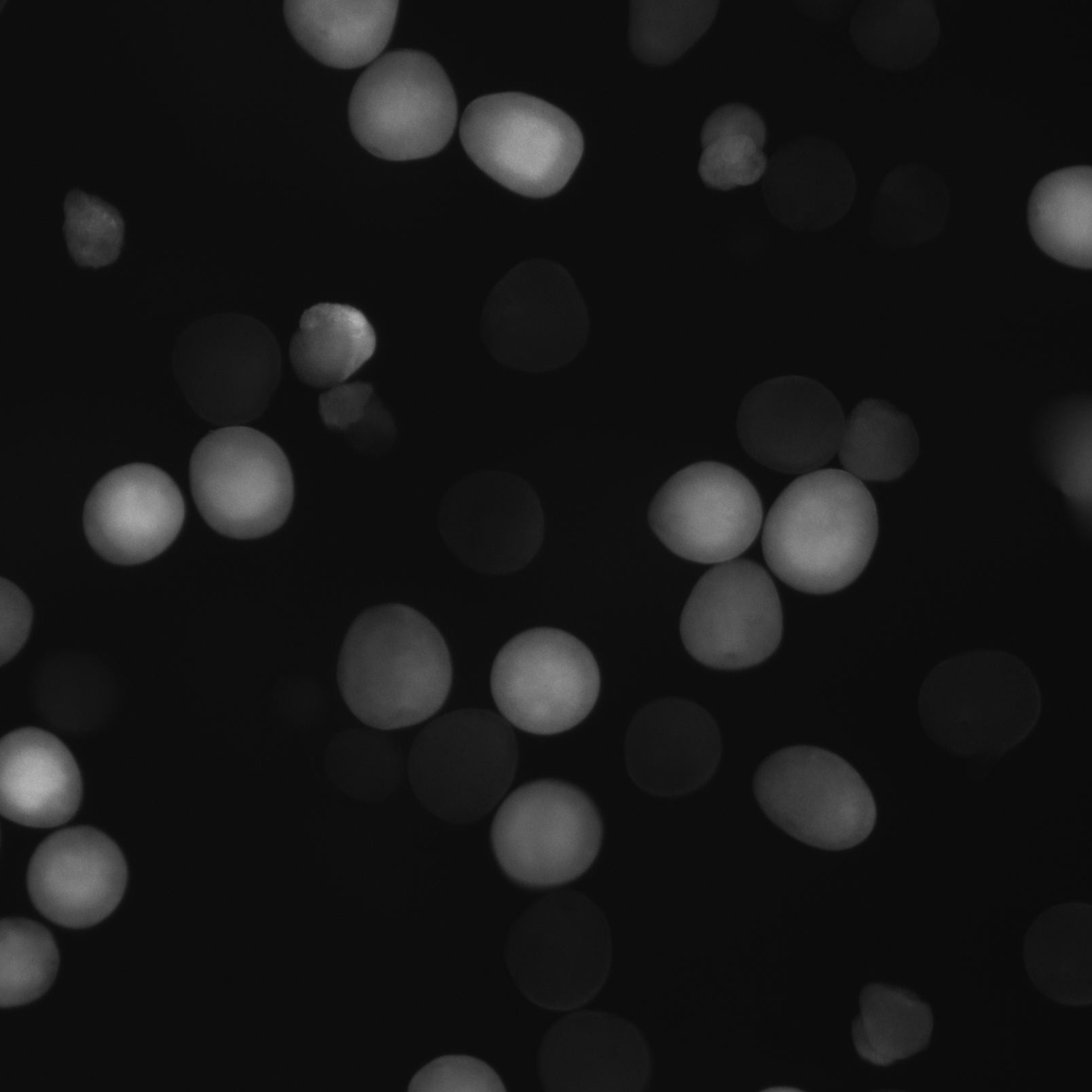

Supplement: Supplementary file 2 — Source data [file 44319_2024_350_MOESM2_ESM.zip › source data/Figure 1/1C/Nematostella transgene + mimiR seed and supplementary matches.tif]

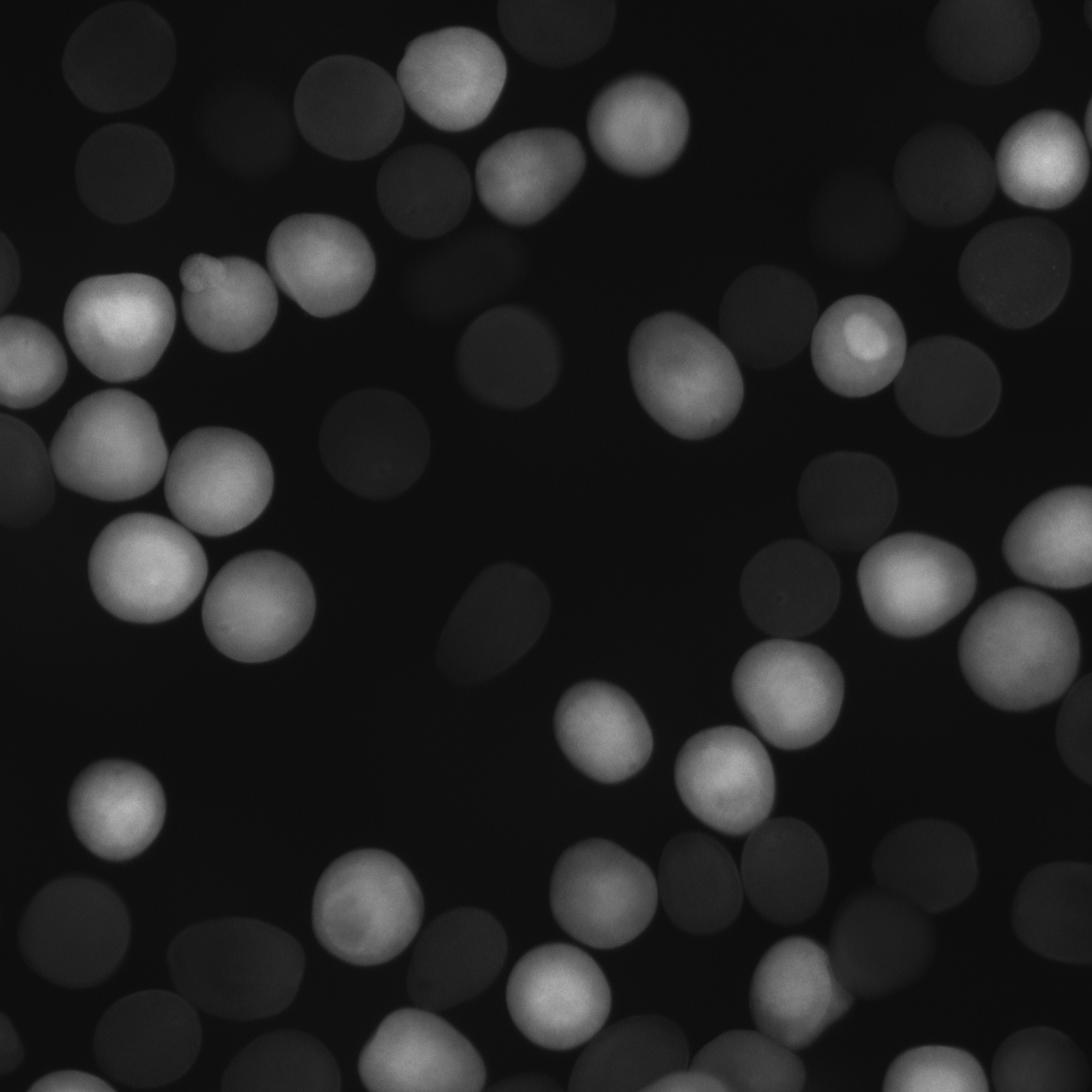

Supplement: Supplementary file 2 — Source data [file 44319_2024_350_MOESM2_ESM.zip › source data/Figure 1/1C/Nematostella transgene + negative control shRNA.tif]

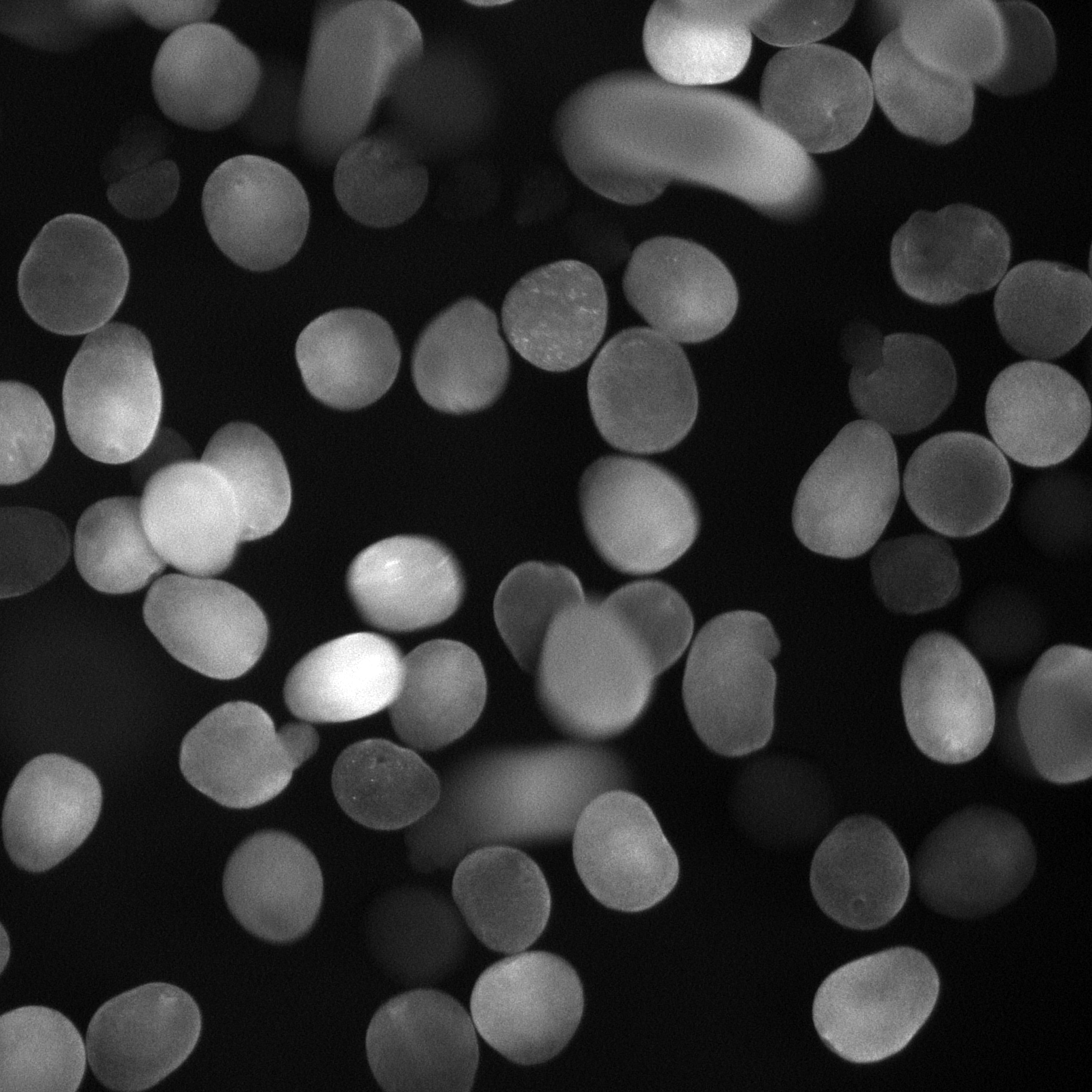

Supplement: Supplementary file 2 — Source data [file 44319_2024_350_MOESM2_ESM.zip › source data/Figure 1/1C/Nematostella transgene + positive control mimiR.tif]

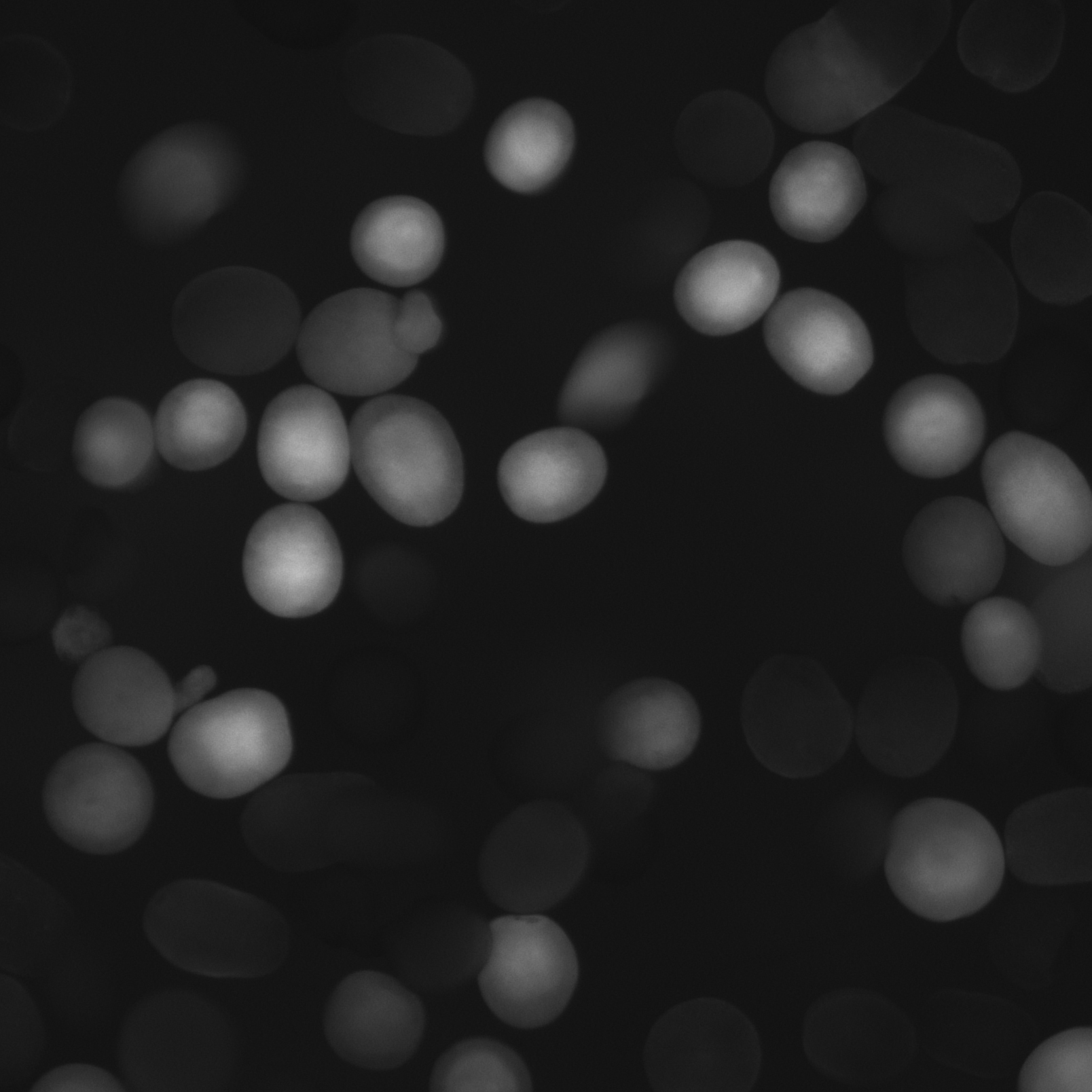

Supplement: Supplementary file 2 — Source data [file 44319_2024_350_MOESM2_ESM.zip › source data/Figure 1/1D/Nematostella transgene + mimiR positions 10-11.tif]

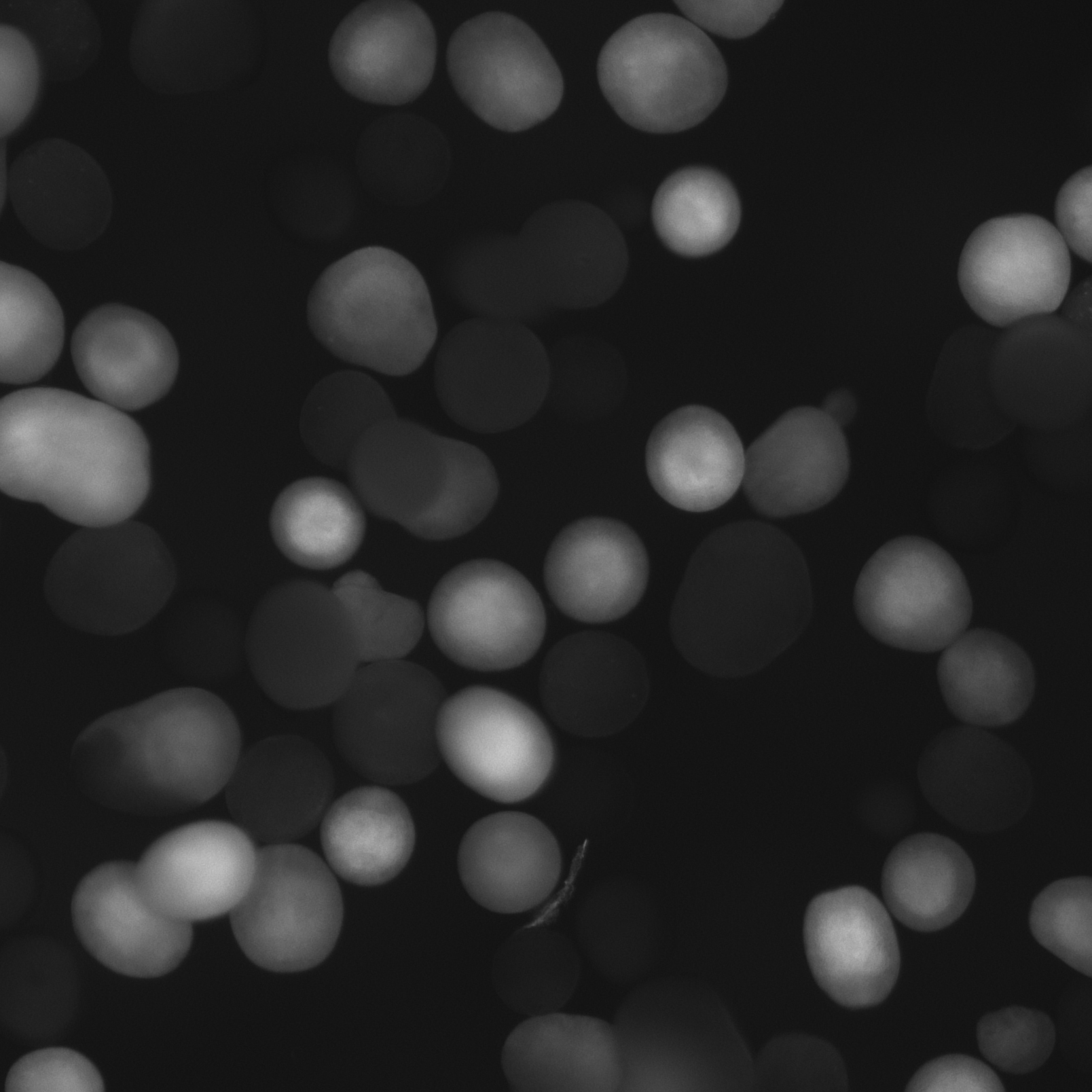

Supplement: Supplementary file 2 — Source data [file 44319_2024_350_MOESM2_ESM.zip › source data/Figure 1/1D/Nematostella transgene + negative control shRNA.tif]

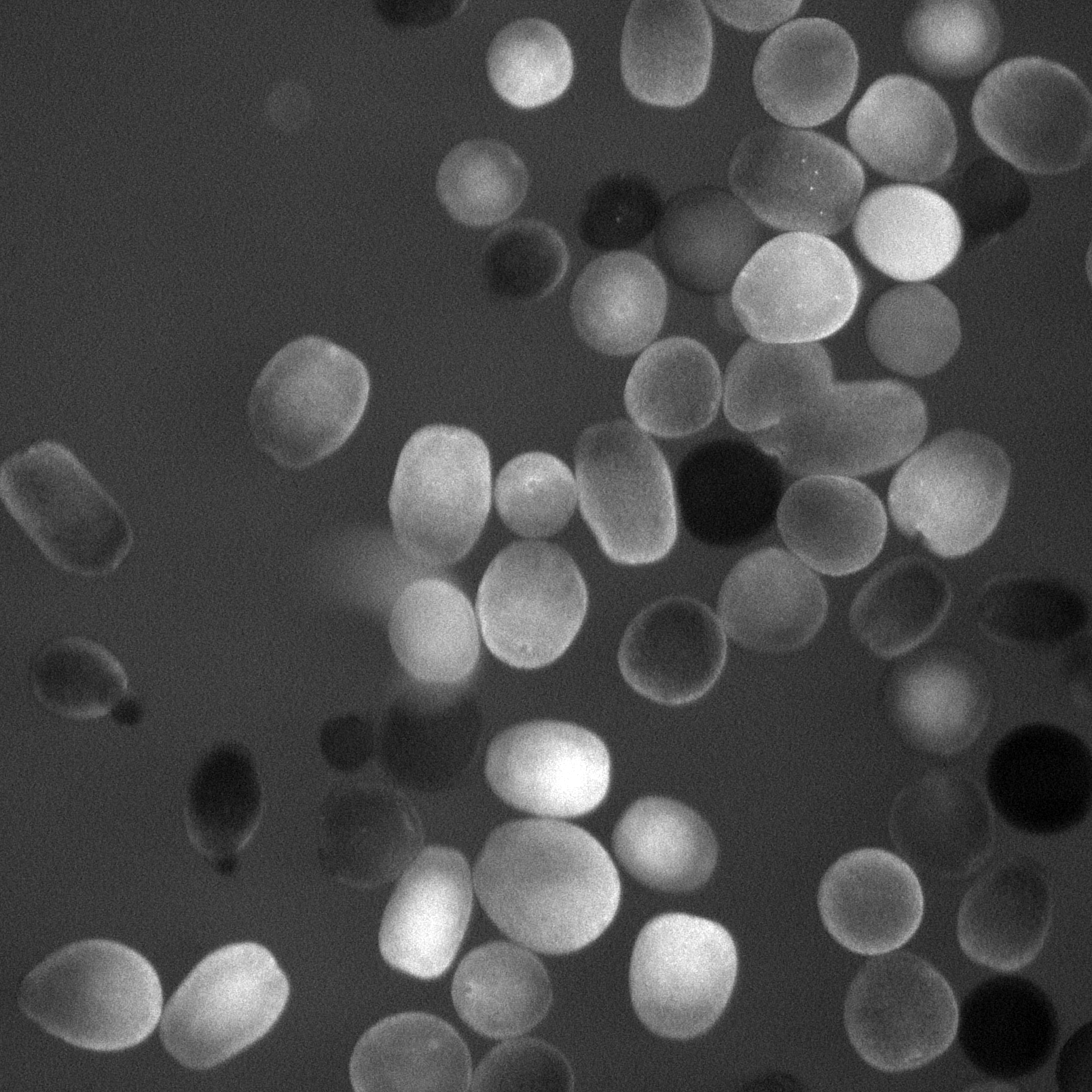

Supplement: Supplementary file 2 — Source data [file 44319_2024_350_MOESM2_ESM.zip › source data/Figure 1/1D/Nematostella transgene + positive control mimiR.tif]

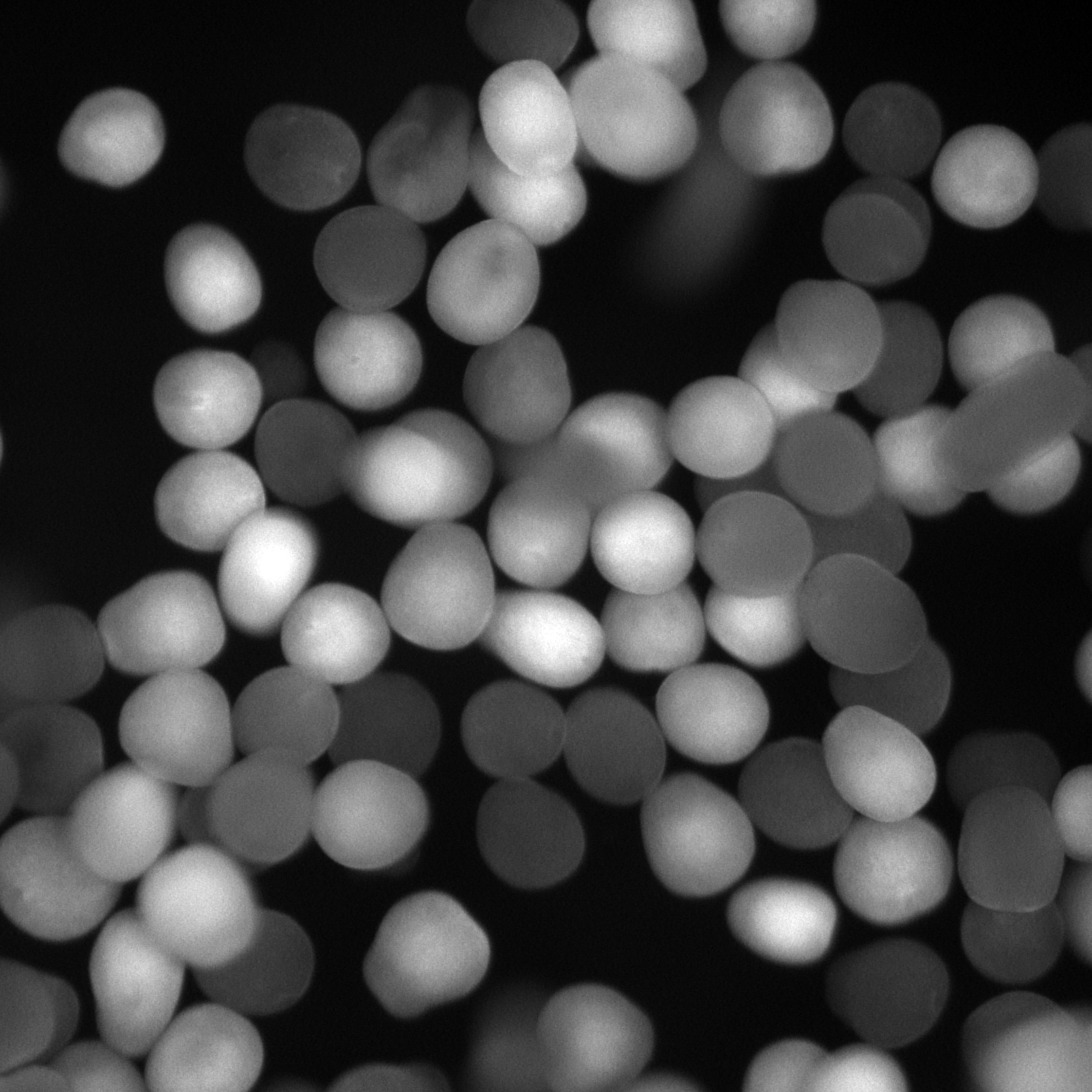

Supplement: Supplementary file 2 — Source data [file 44319_2024_350_MOESM2_ESM.zip › source data/Figure 1/1E/Nematostella transgene + mimiR posiiton 10.tif]

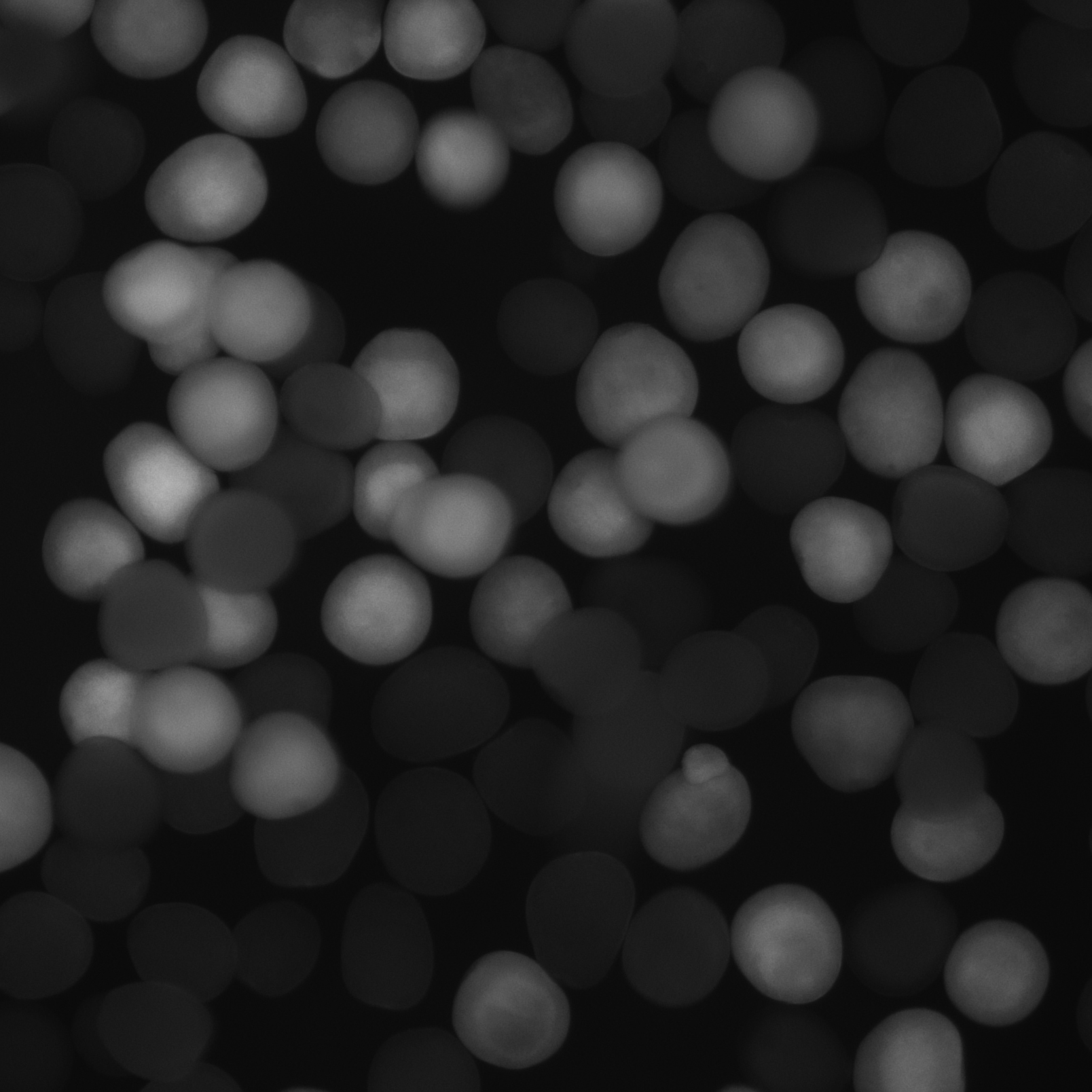

Supplement: Supplementary file 2 — Source data [file 44319_2024_350_MOESM2_ESM.zip › source data/Figure 1/1E/Nematostella transgene + negative control shRNA.tif]

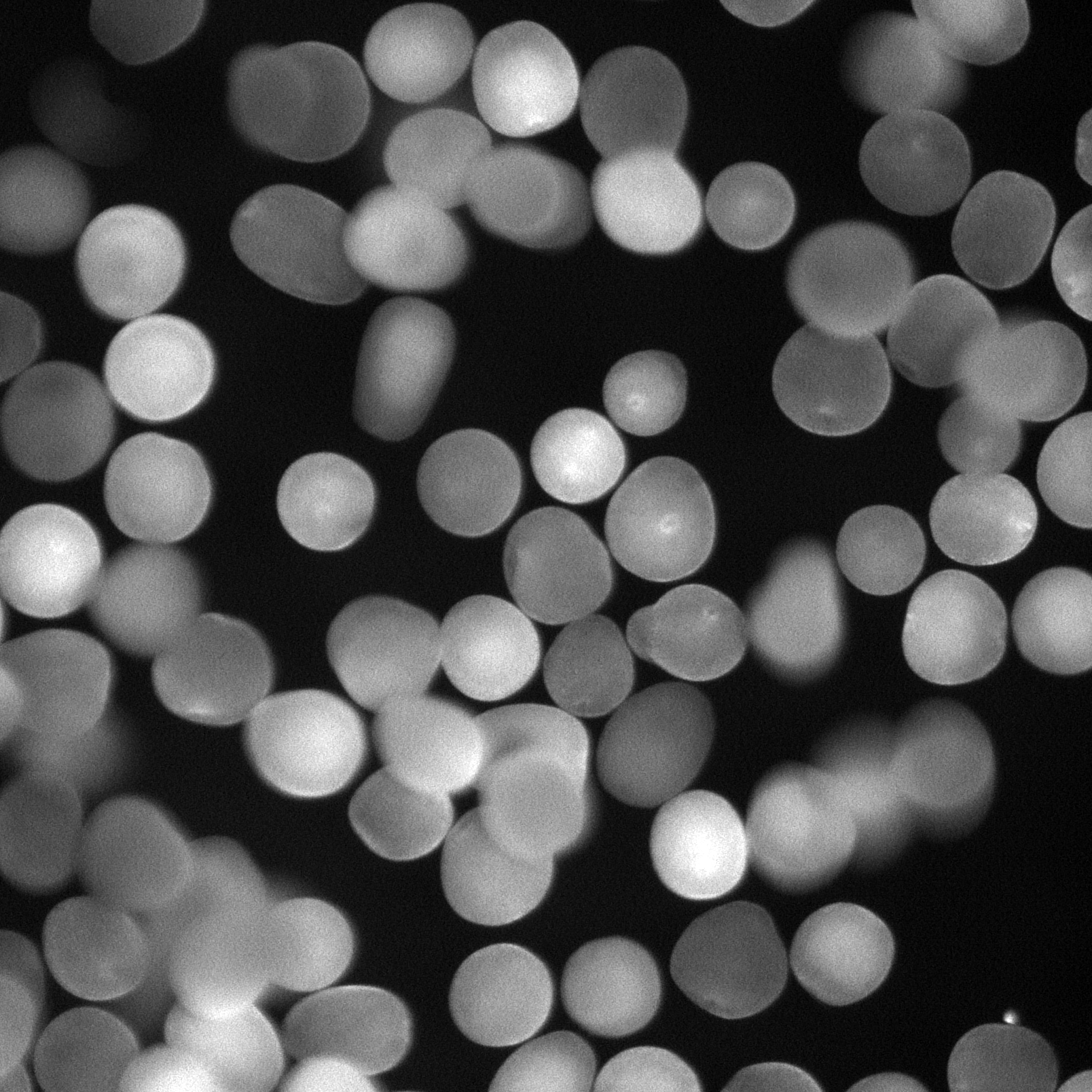

Supplement: Supplementary file 2 — Source data [file 44319_2024_350_MOESM2_ESM.zip › source data/Figure 1/1E/Nematostella transgene + positive control mimiR.tif]

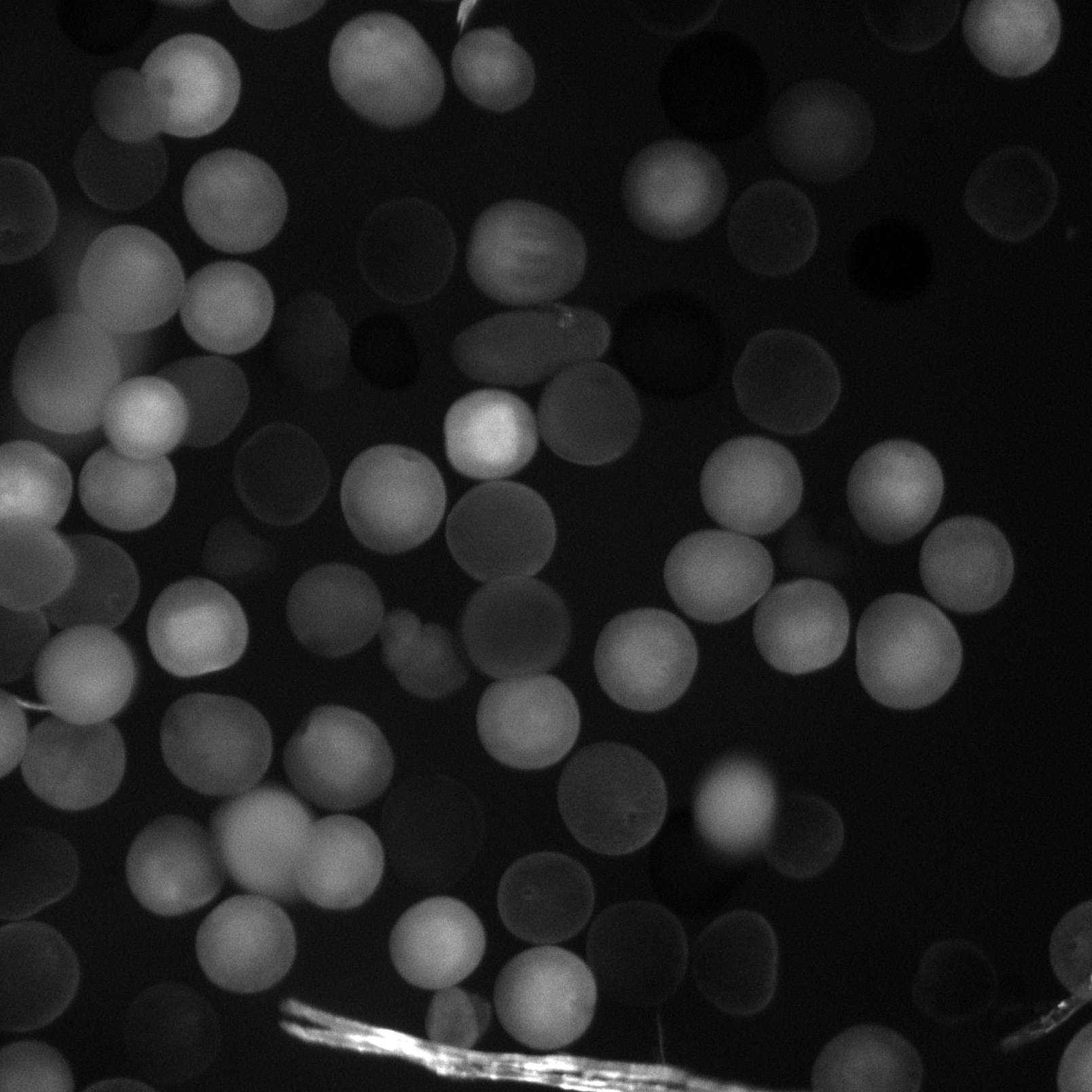

Supplement: Supplementary file 2 — Source data [file 44319_2024_350_MOESM2_ESM.zip › source data/Figure 1/1F/Nematostella transgene + mimiR position 11.tif]

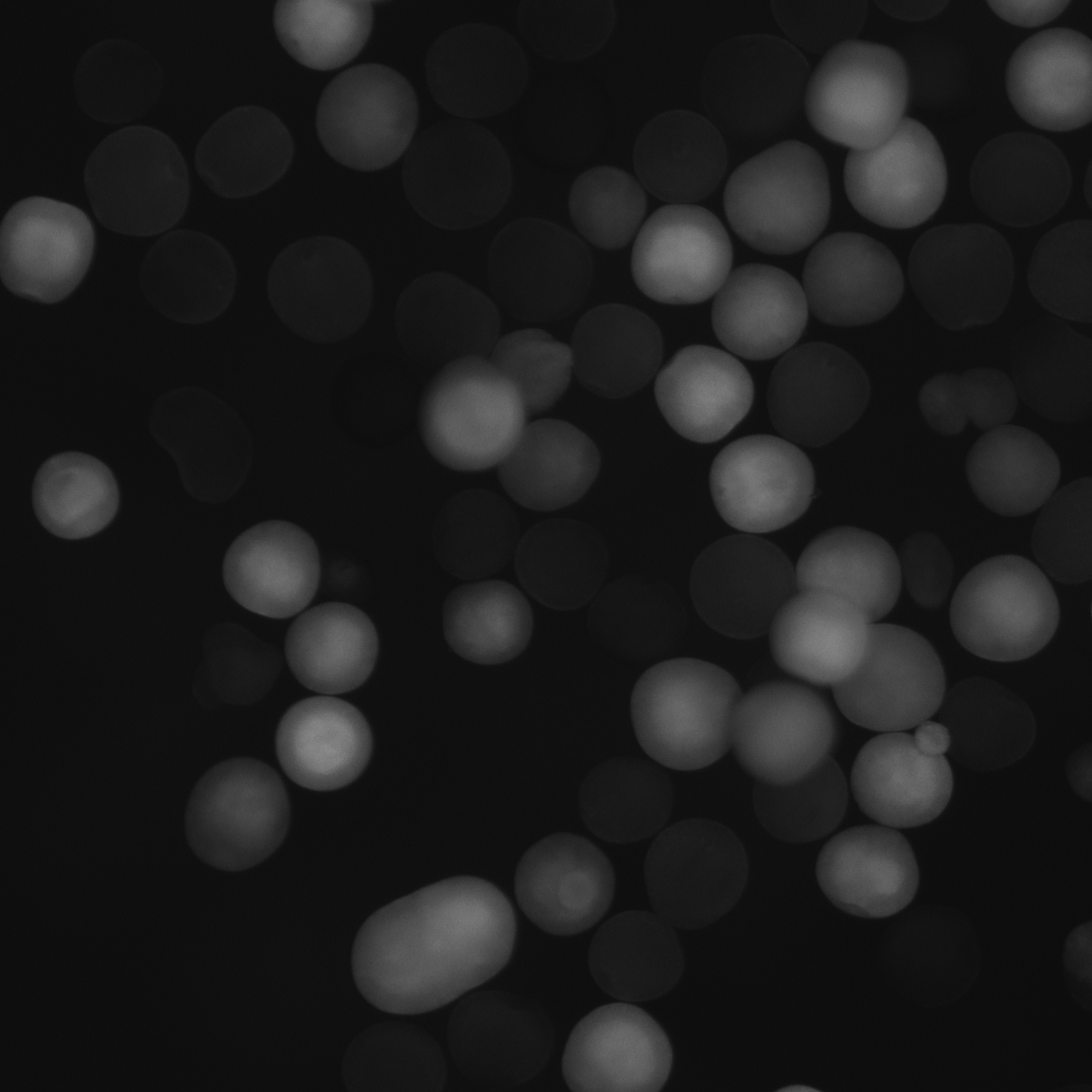

Supplement: Supplementary file 2 — Source data [file 44319_2024_350_MOESM2_ESM.zip › source data/Figure 1/1F/Nematostella transgene + negative control shRNA.tif]

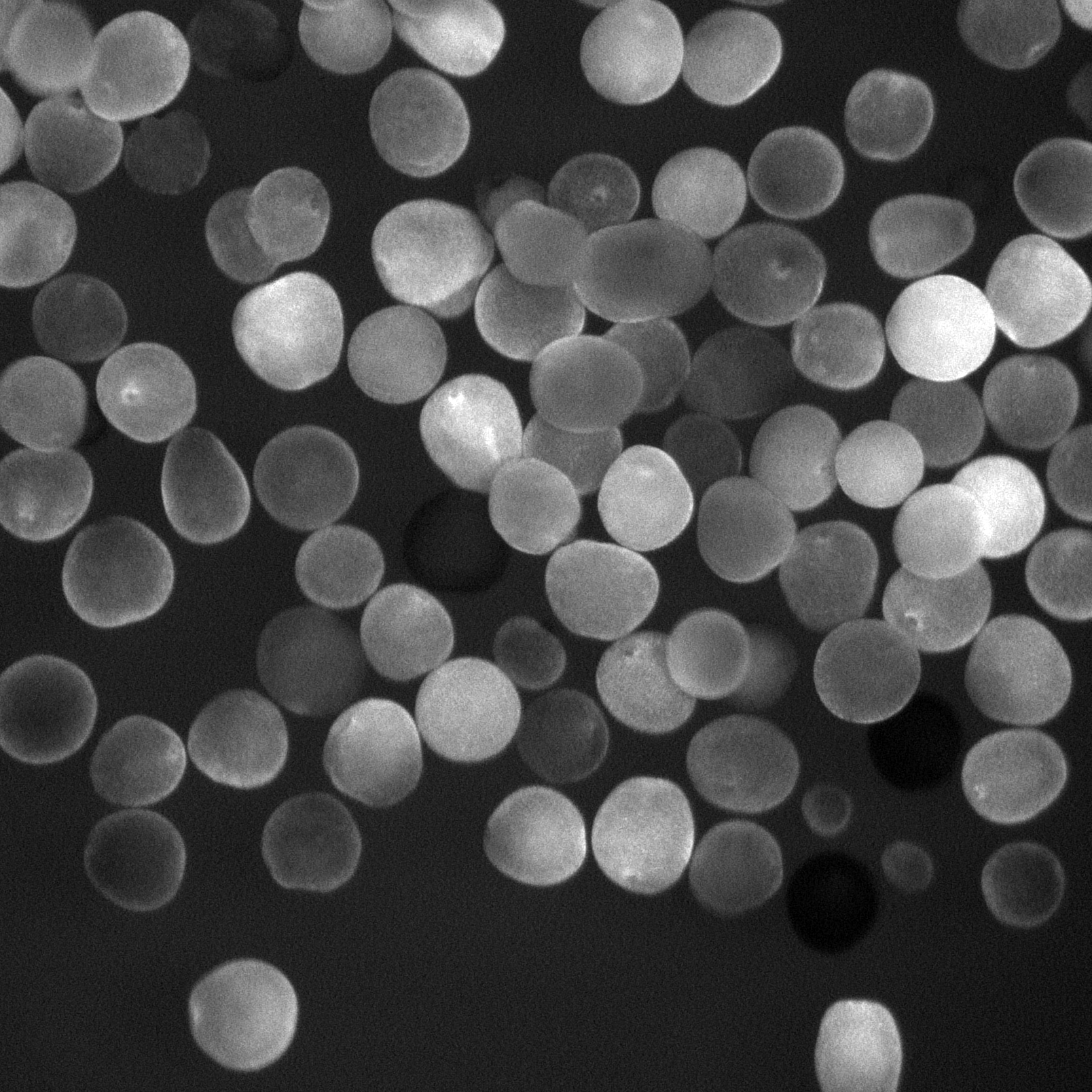

Supplement: Supplementary file 2 — Source data [file 44319_2024_350_MOESM2_ESM.zip › source data/Figure 1/1F/Nematostella transgene + positive control mimiR.tif]

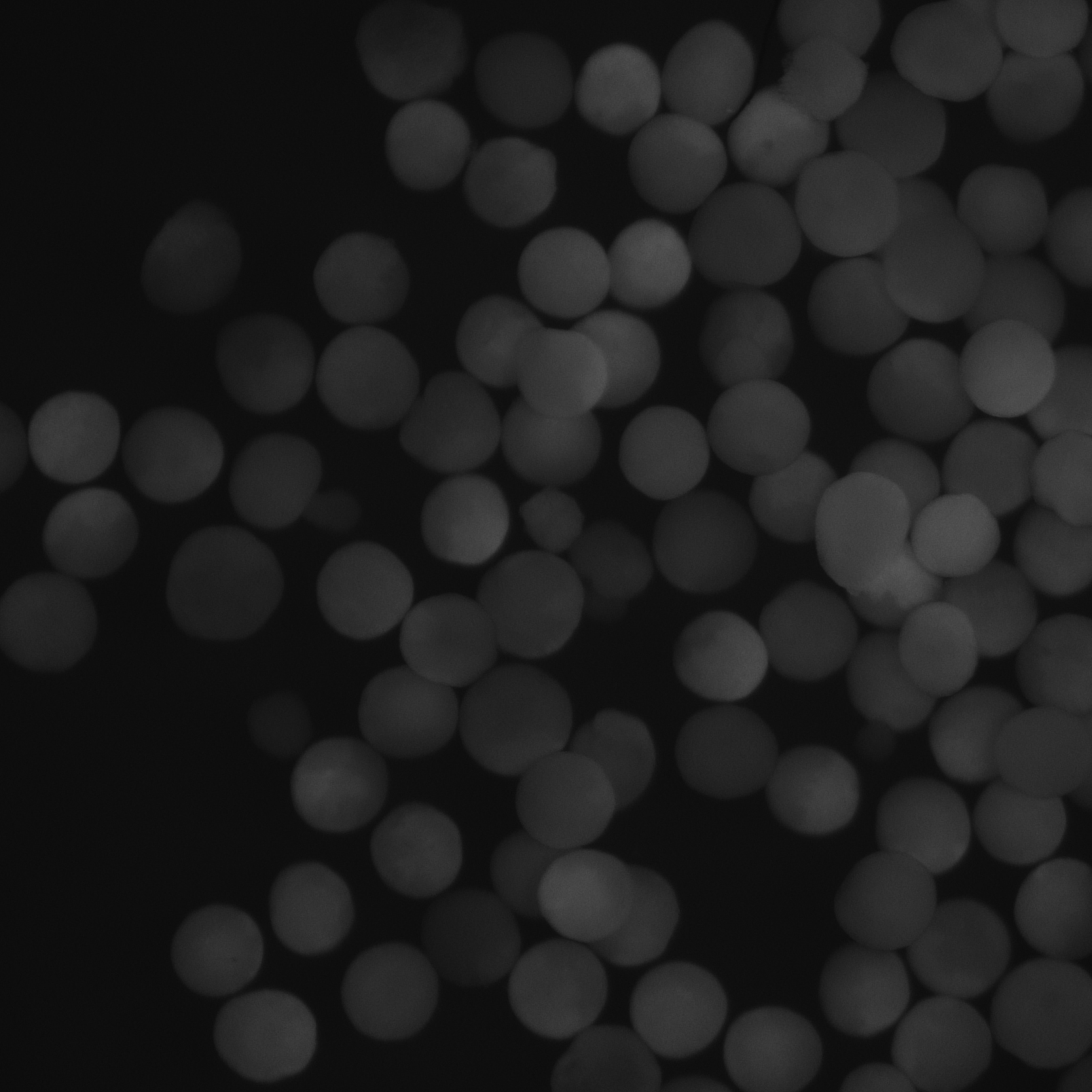

Supplement: Supplementary file 2 — Source data [file 44319_2024_350_MOESM2_ESM.zip › source data/Figure 2/2B/Nematostella mRNA + mimiR seed.tif]

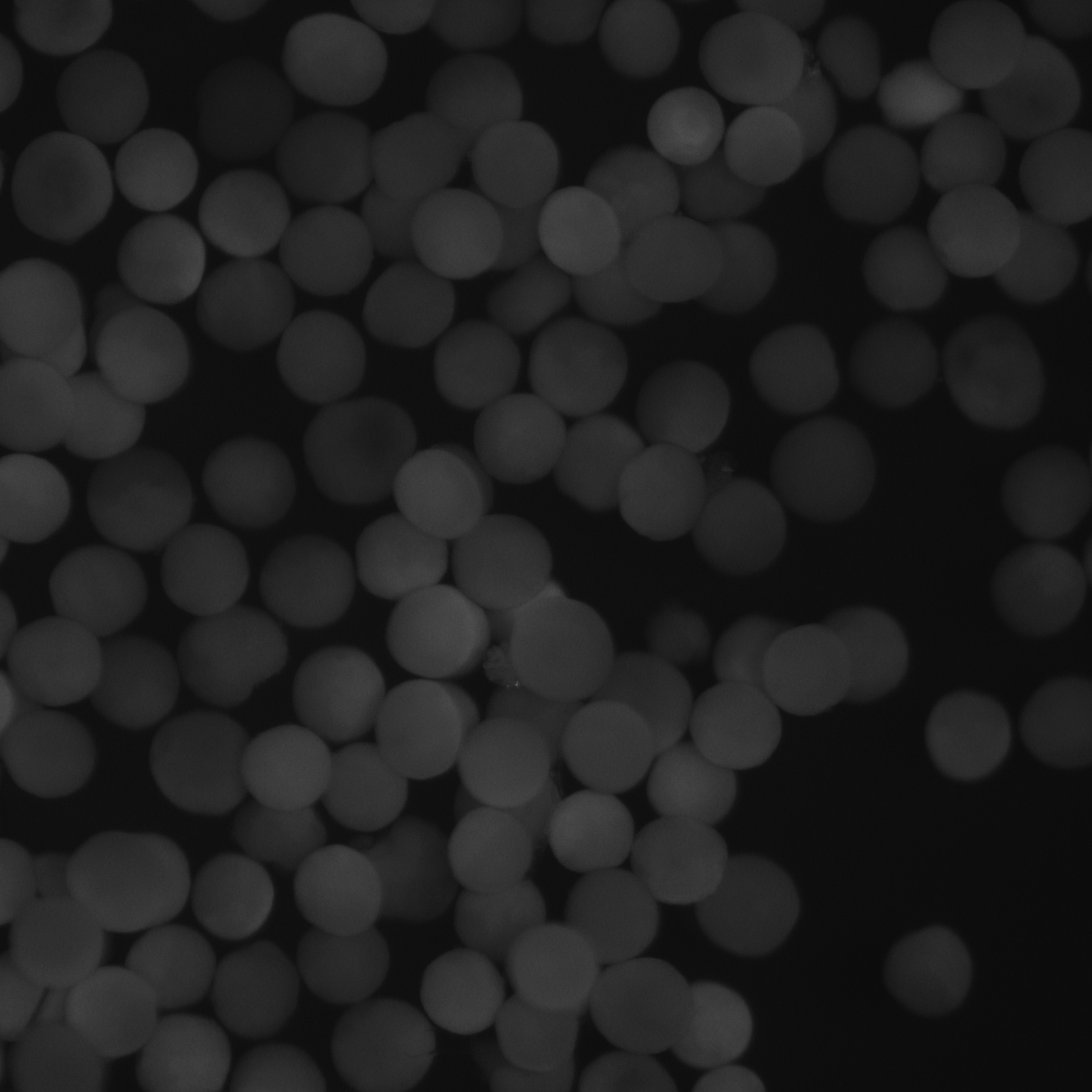

Supplement: Supplementary file 2 — Source data [file 44319_2024_350_MOESM2_ESM.zip › source data/Figure 2/2B/Nematostella mRNA + negative control shRNA.tif]

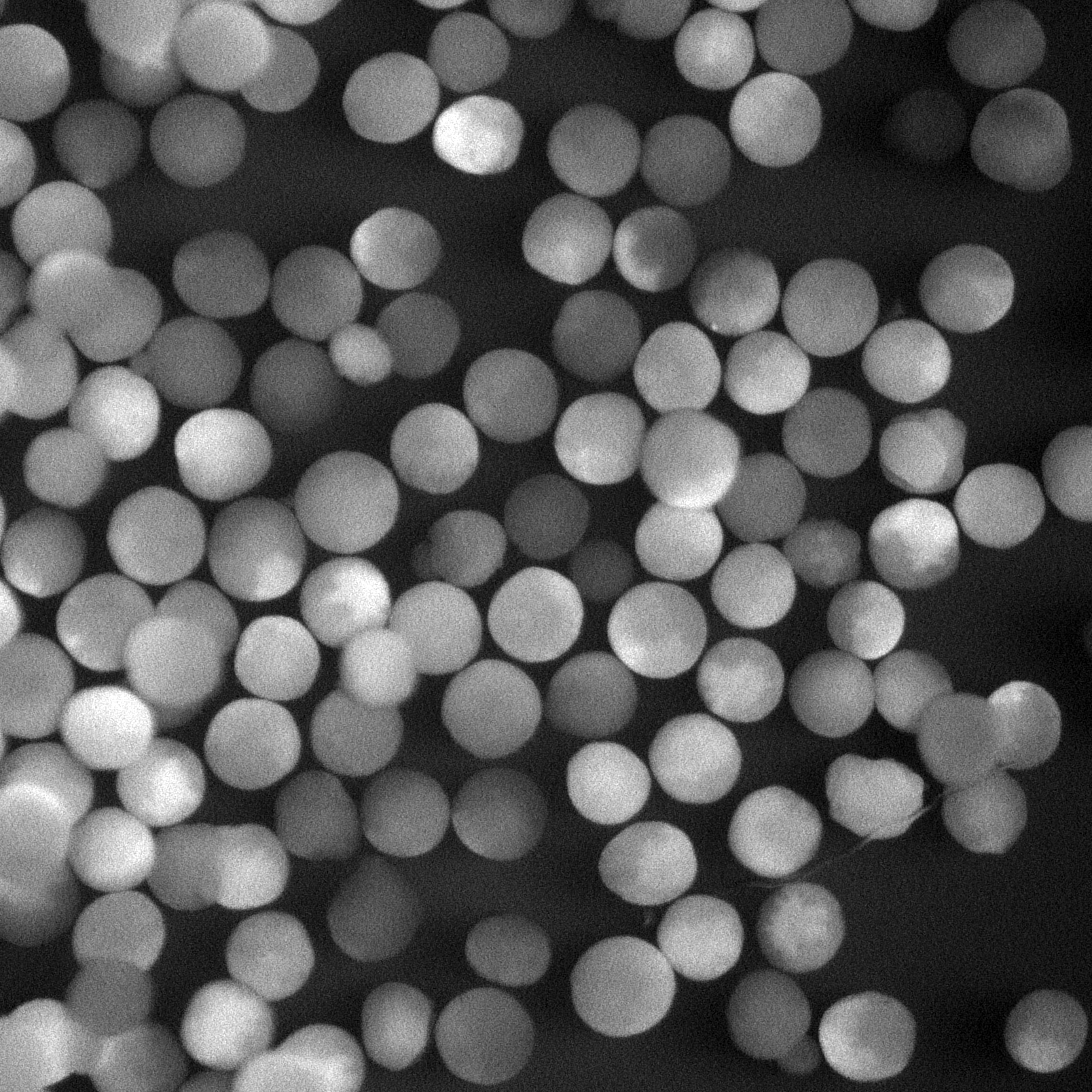

Supplement: Supplementary file 2 — Source data [file 44319_2024_350_MOESM2_ESM.zip › source data/Figure 2/2B/Nematostella mRNA + positive control mimiR.tif]

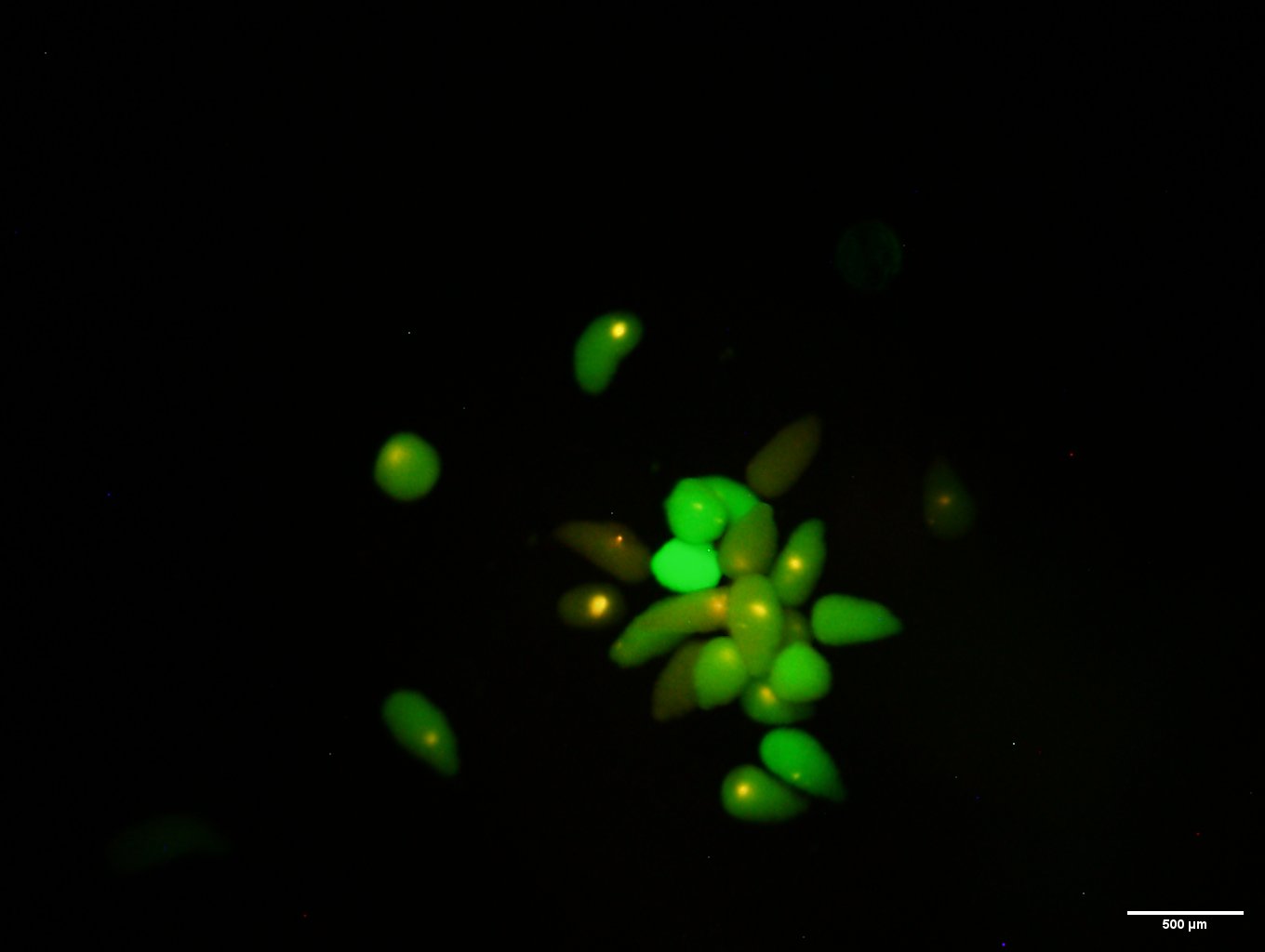

Supplement: Supplementary file 2 — Source data [file 44319_2024_350_MOESM2_ESM.zip › source data/Figure 3/3A/Hydractinia + mimiR seed_green filter.jpg]

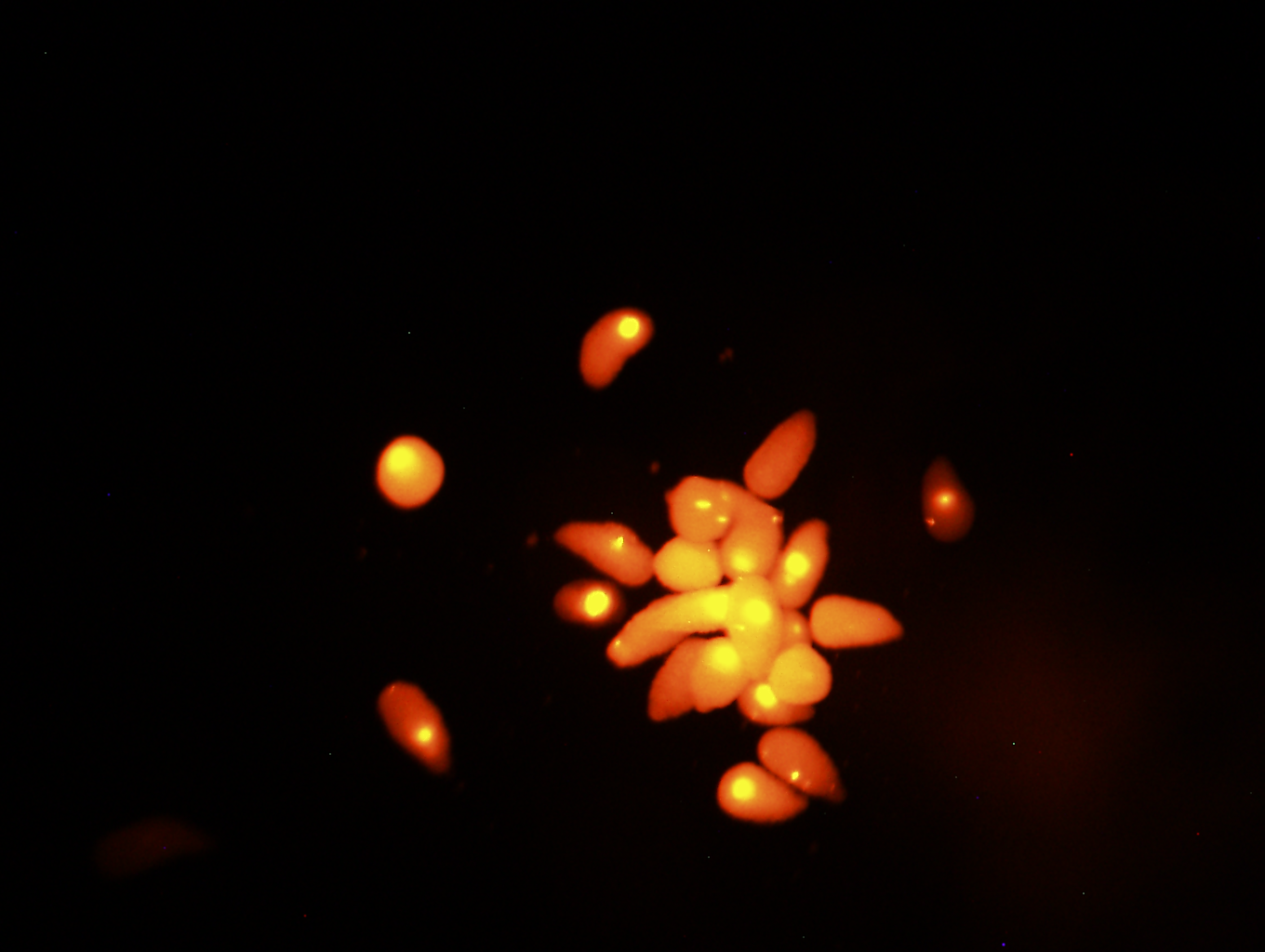

Supplement: Supplementary file 2 — Source data [file 44319_2024_350_MOESM2_ESM.zip › source data/Figure 3/3A/Hydractinia + mimiR seed_red filter.tif]

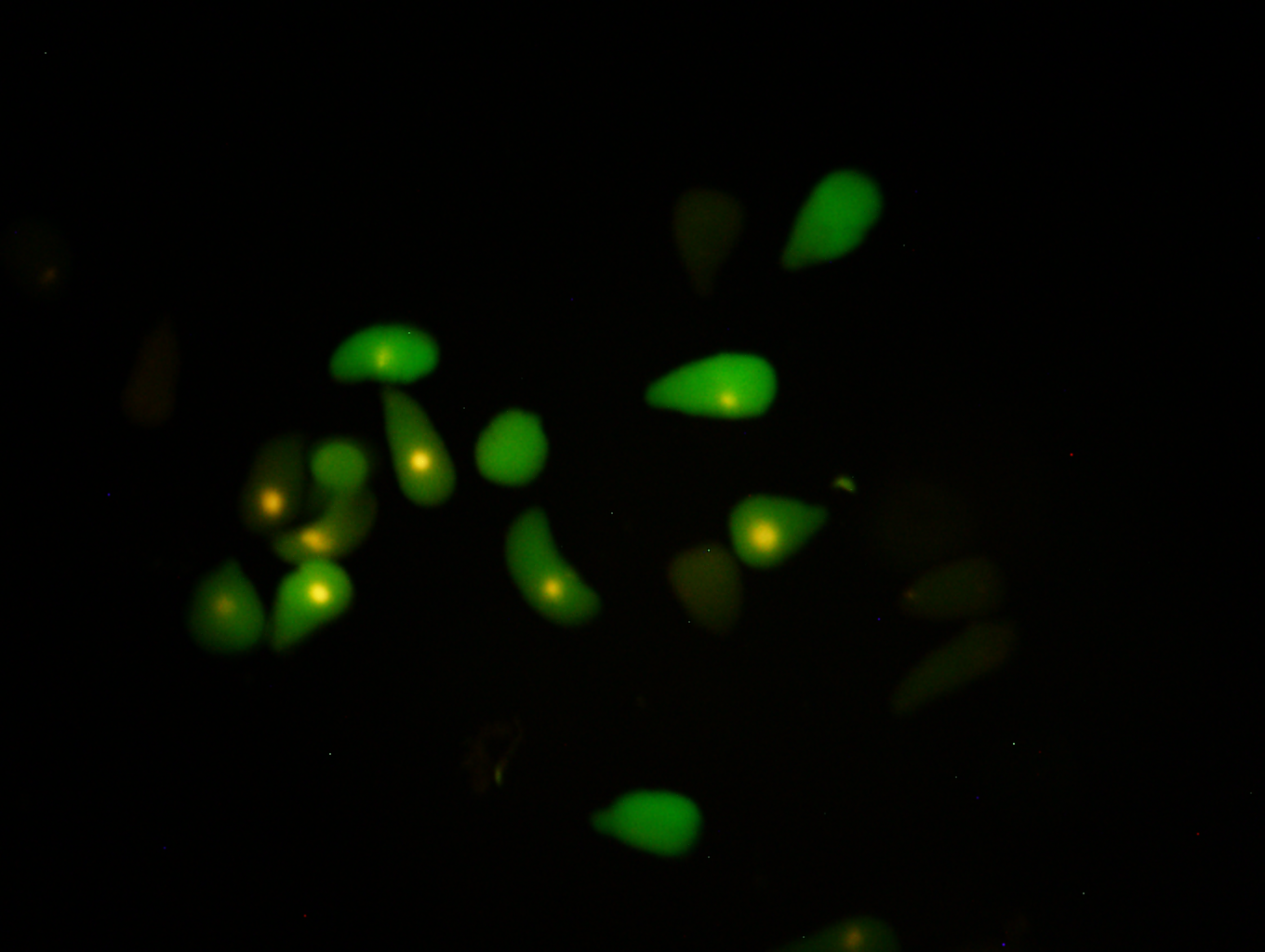

Supplement: Supplementary file 2 — Source data [file 44319_2024_350_MOESM2_ESM.zip › source data/Figure 3/3A/Hydractinia + Negative Control shRNA_green filter.tif]

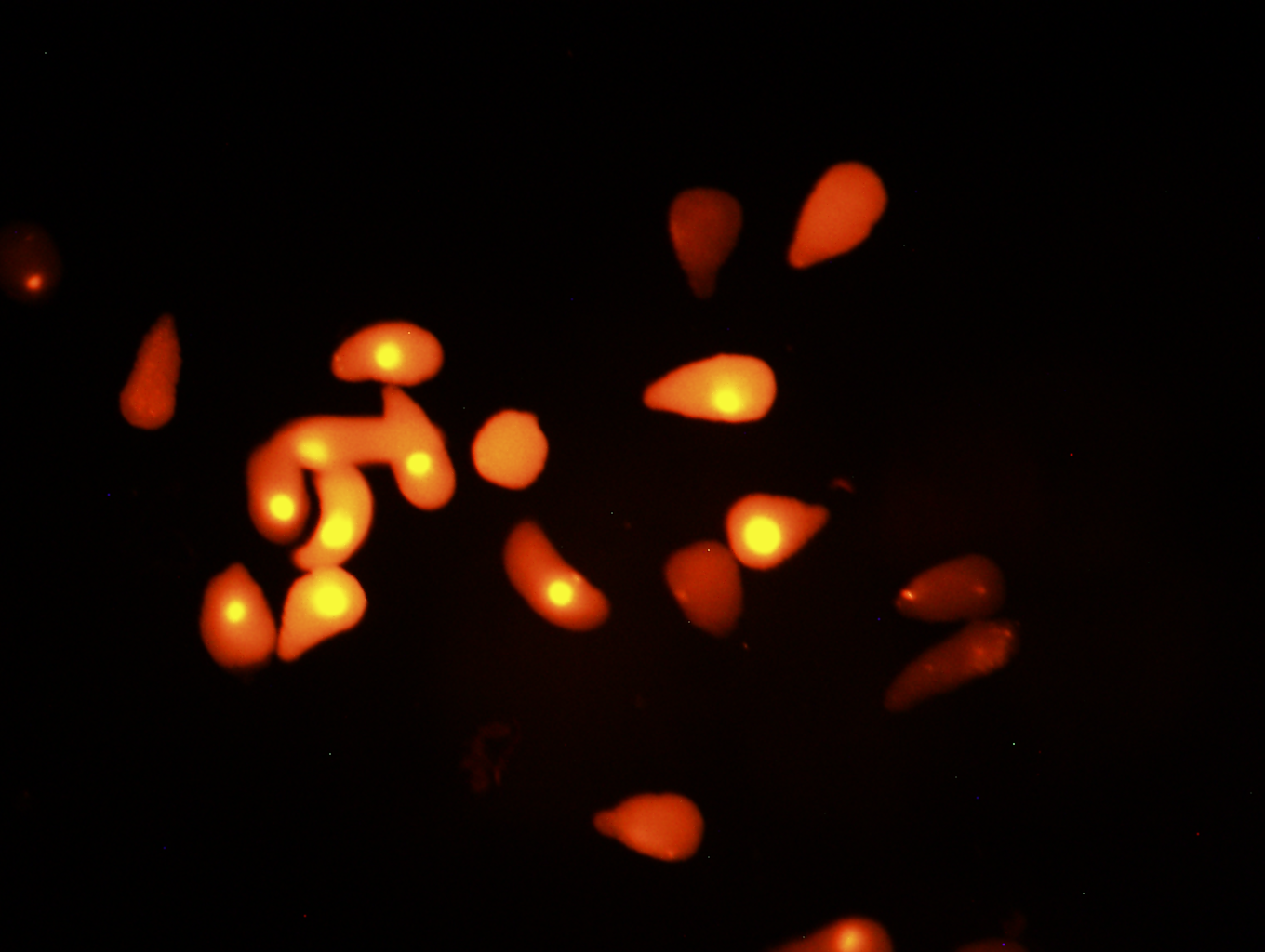

Supplement: Supplementary file 2 — Source data [file 44319_2024_350_MOESM2_ESM.zip › source data/Figure 3/3A/Hydractinia + Negative Control shRNA_red filter.tif]

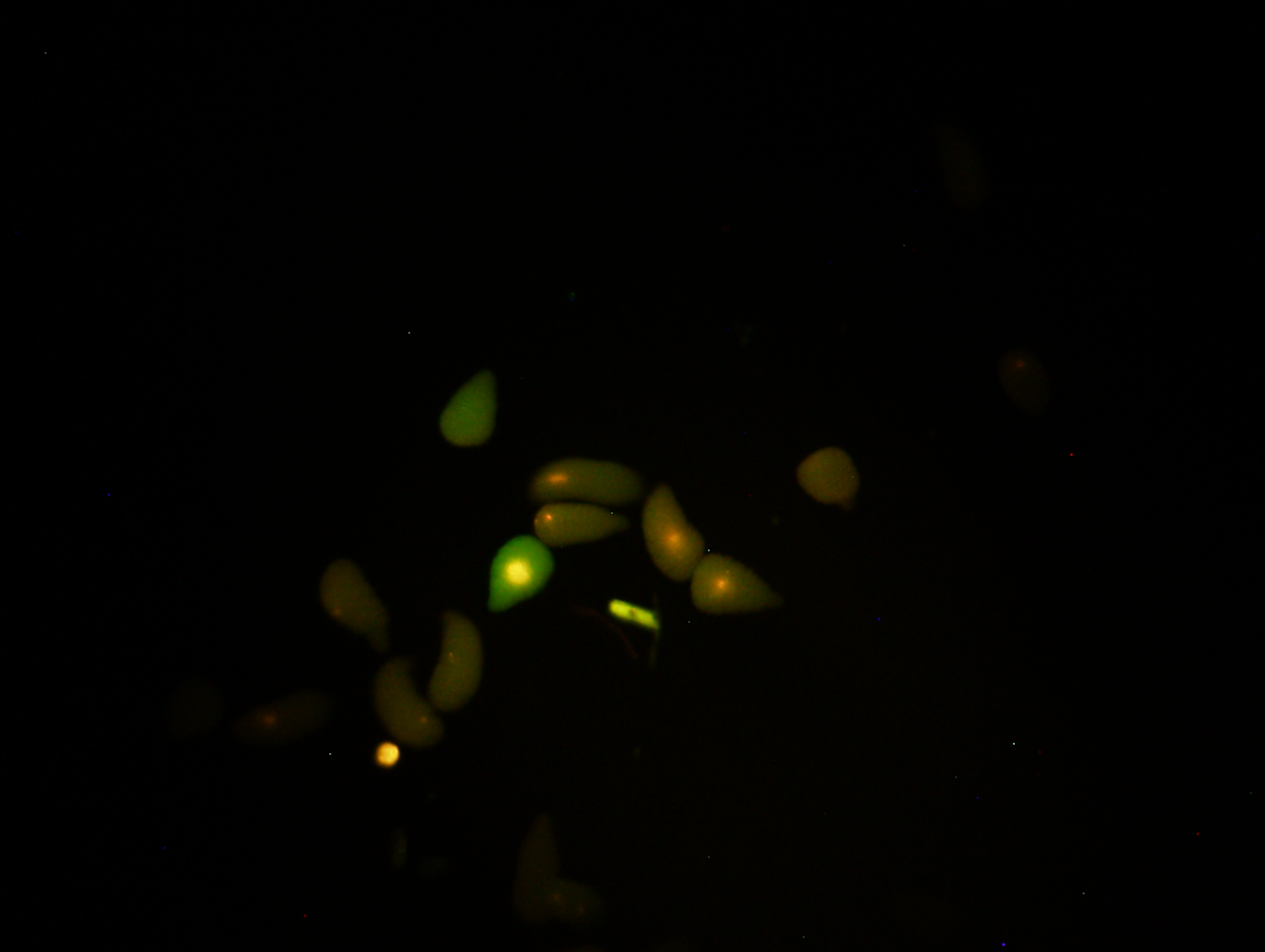

Supplement: Supplementary file 2 — Source data [file 44319_2024_350_MOESM2_ESM.zip › source data/Figure 3/3A/Hydractinia + Positive Control mimiR_green filter.tif]

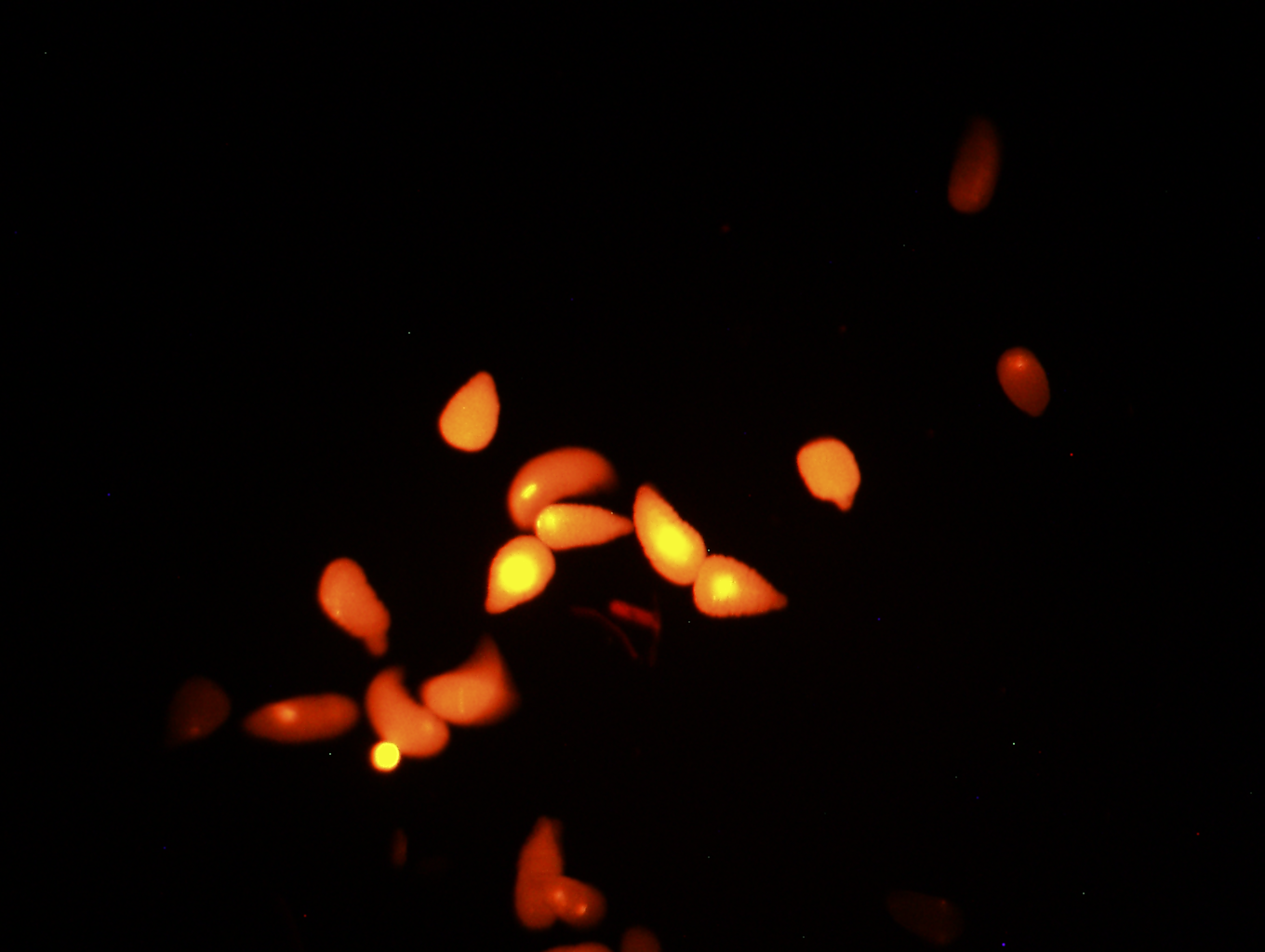

Supplement: Supplementary file 2 — Source data [file 44319_2024_350_MOESM2_ESM.zip › source data/Figure 3/3A/Hydractinia + Positive Control mimiR_red filter.tif]

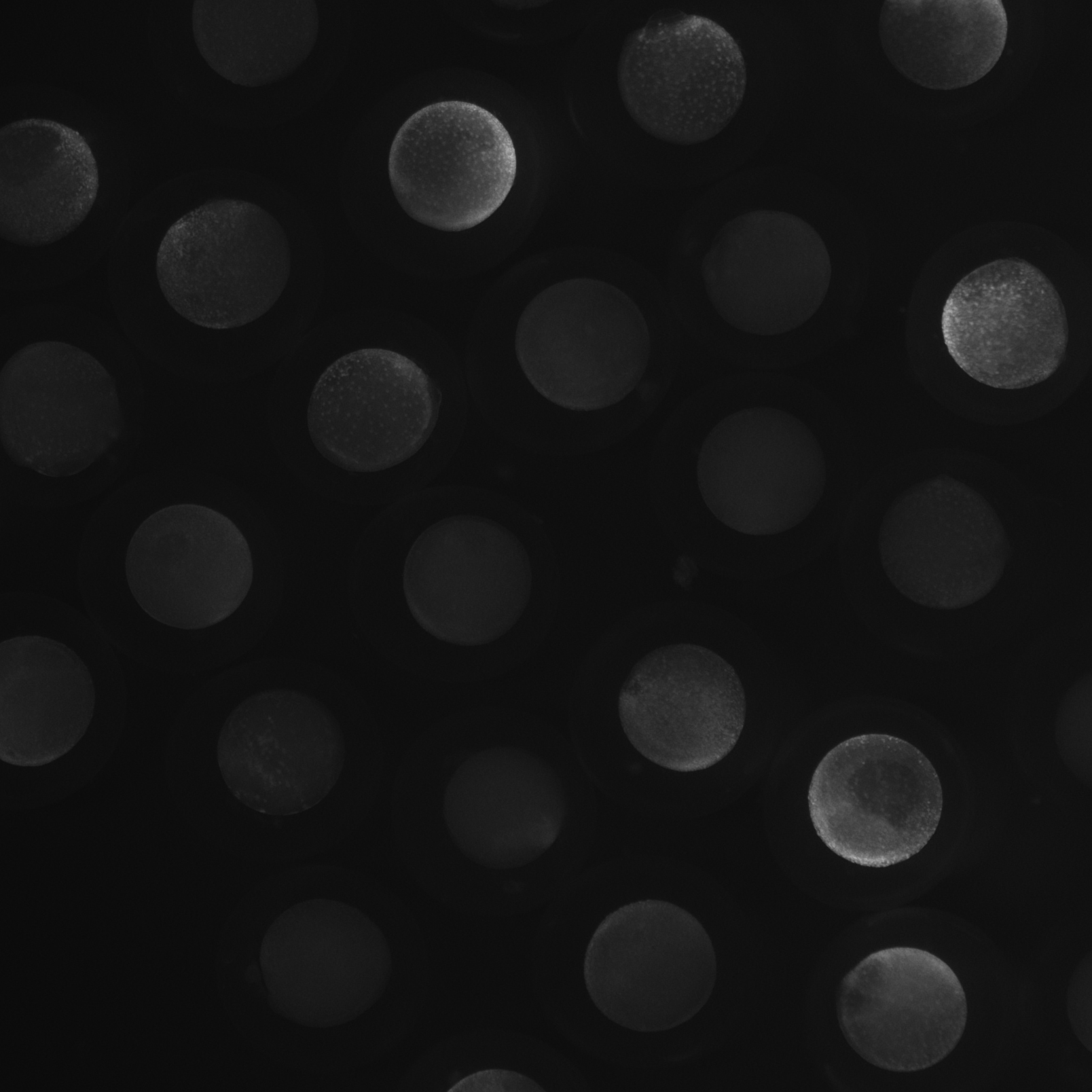

Supplement: Supplementary file 2 — Source data [file 44319_2024_350_MOESM2_ESM.zip › source data/Figure 5/5A/Zebrafish + mimiR seed_GFP filter.tif]

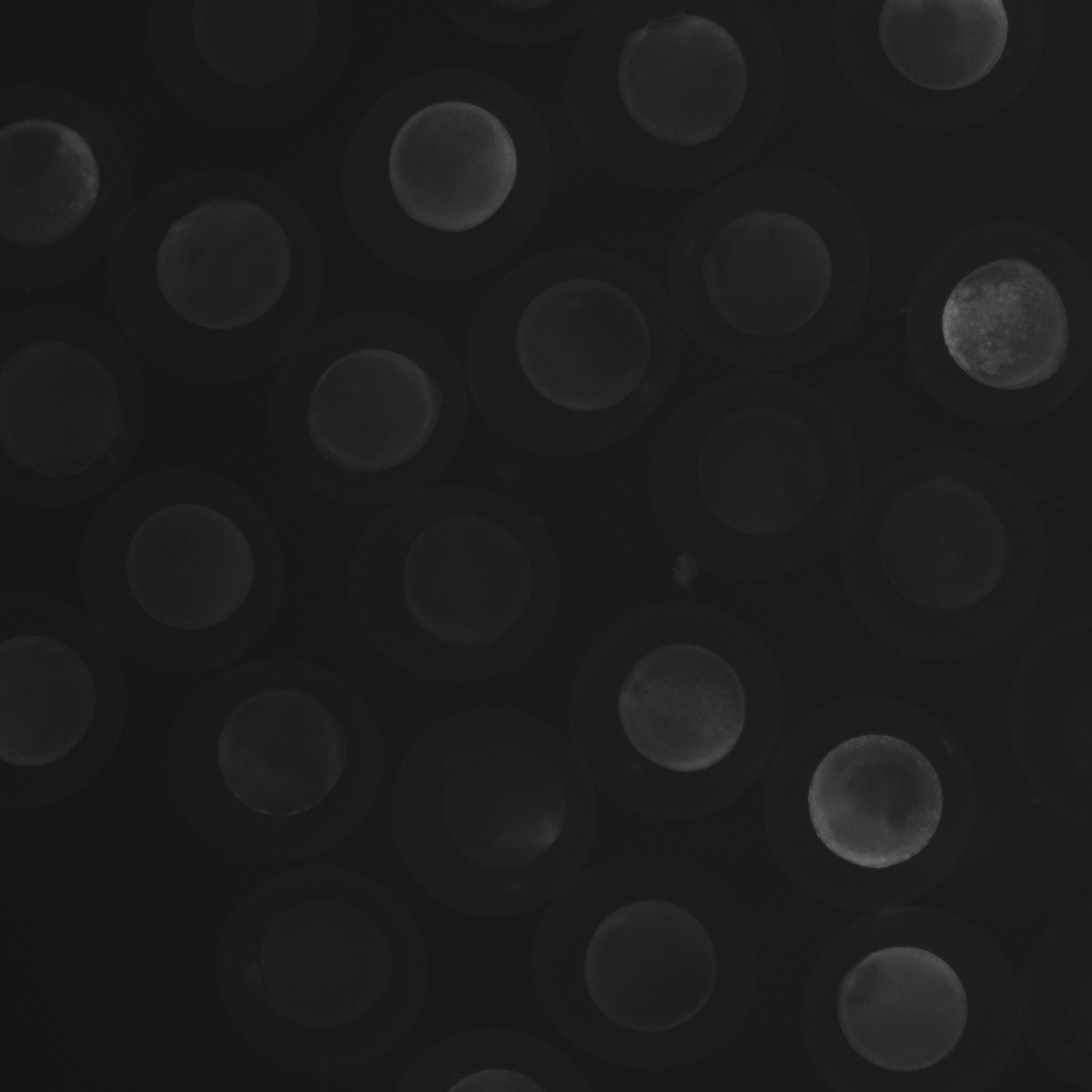

Supplement: Supplementary file 2 — Source data [file 44319_2024_350_MOESM2_ESM.zip › source data/Figure 5/5A/Zebrafish + mimiR seed_mCherry filter.tif]

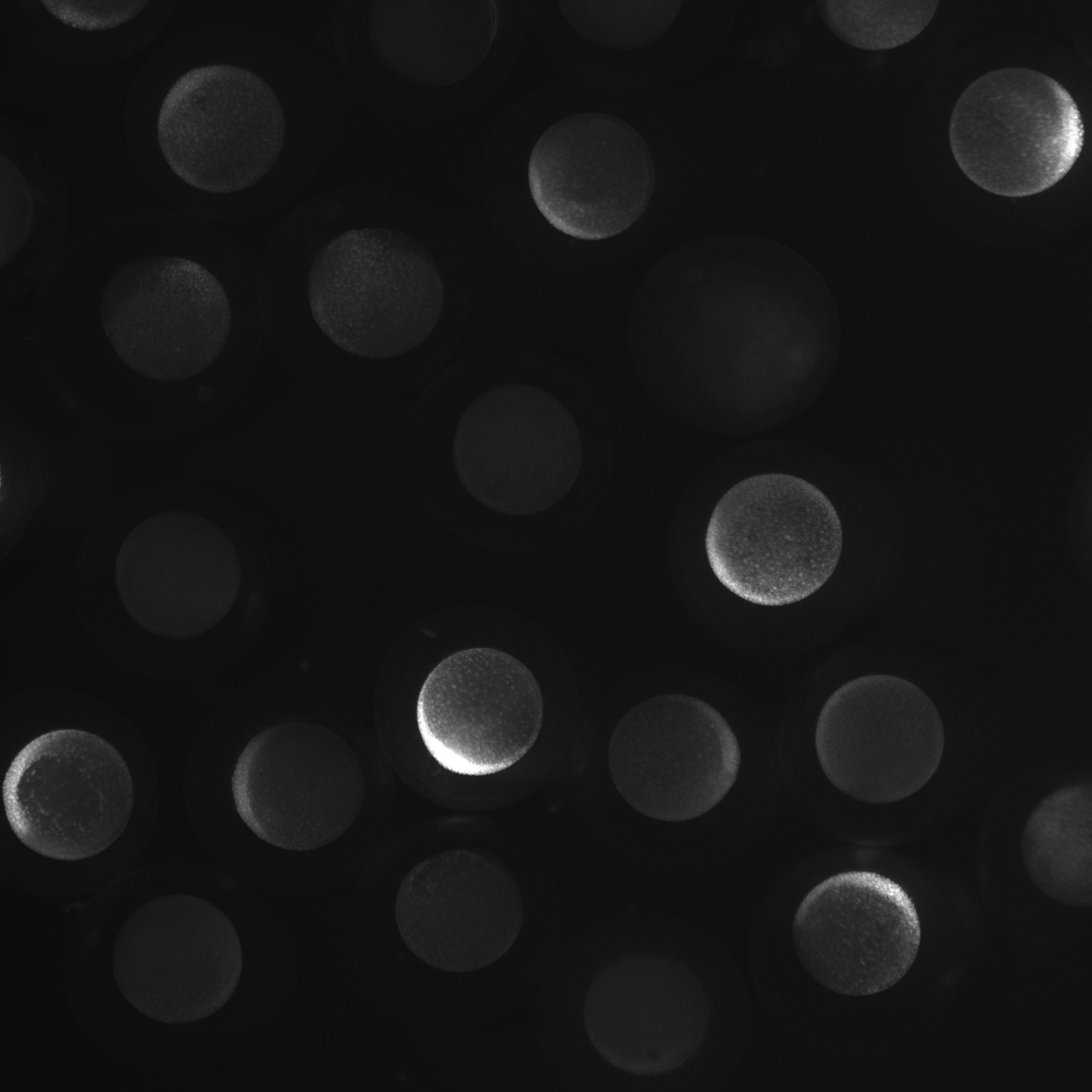

Supplement: Supplementary file 2 — Source data [file 44319_2024_350_MOESM2_ESM.zip › source data/Figure 5/5A/Zebrafish + Negative Control shRNA_GFP filter.tif]

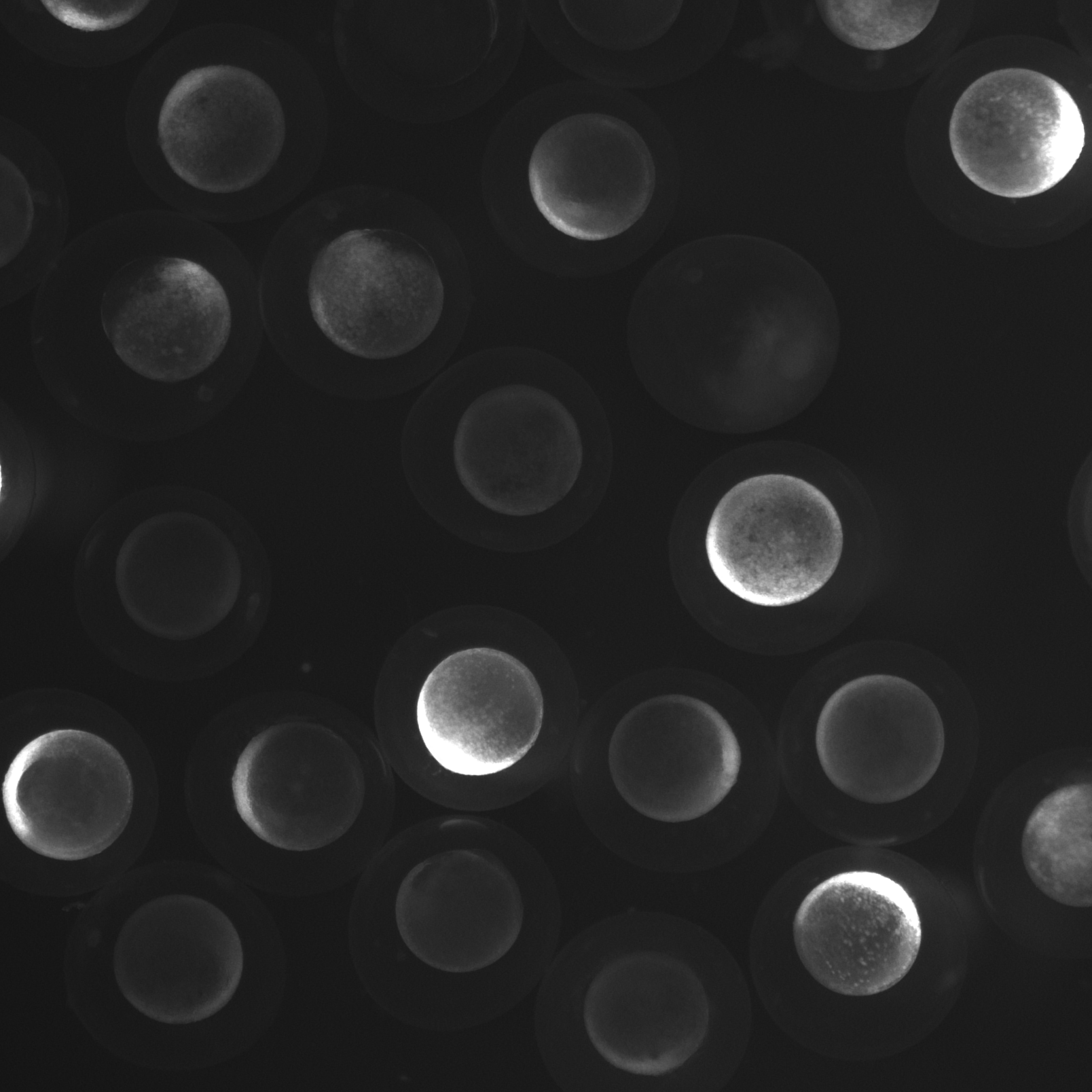

Supplement: Supplementary file 2 — Source data [file 44319_2024_350_MOESM2_ESM.zip › source data/Figure 5/5A/Zebrafish + Negative Control shRNA_mCherry filter.tif]

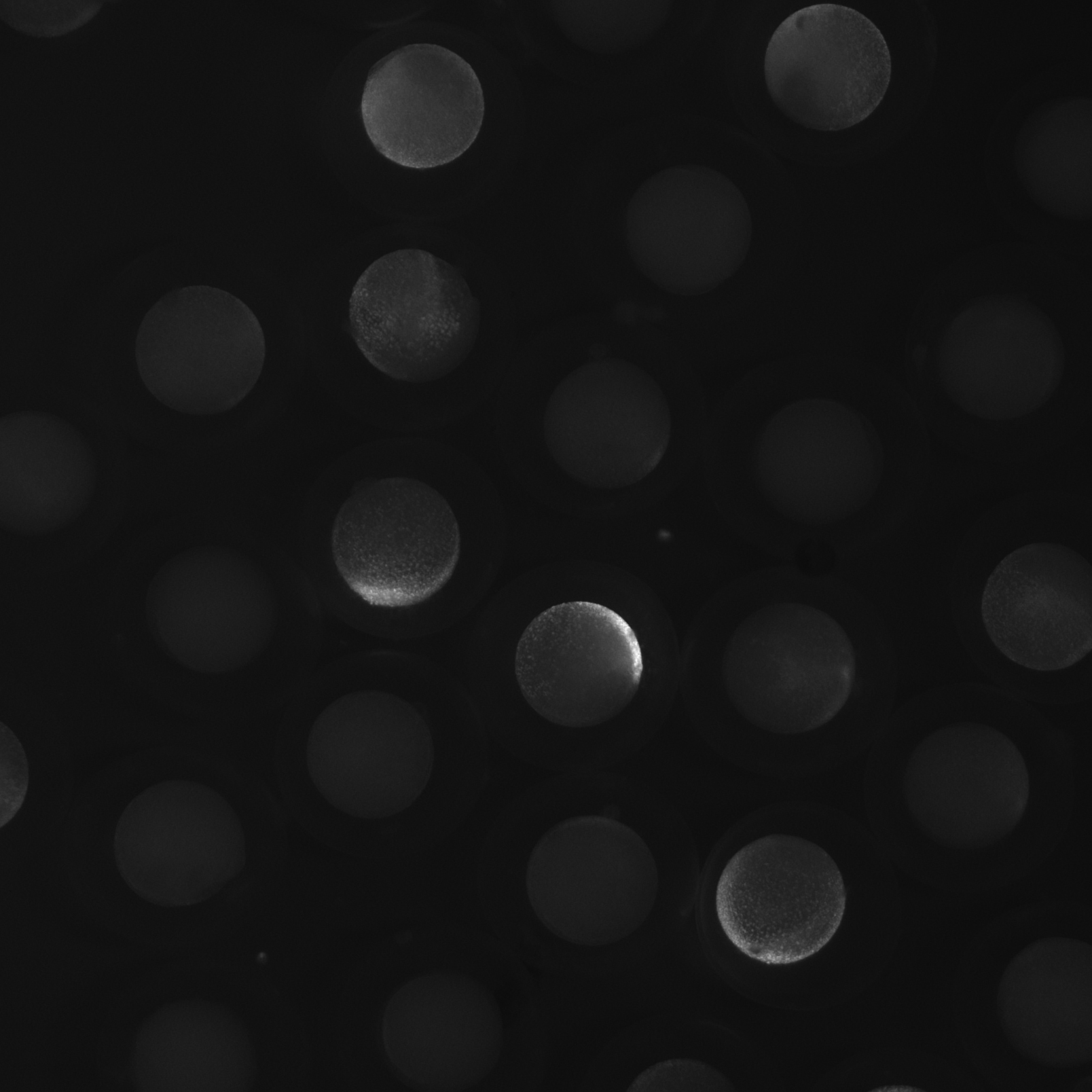

Supplement: Supplementary file 2 — Source data [file 44319_2024_350_MOESM2_ESM.zip › source data/Figure 5/5A/Zebrafish + Positive Control mimiR_GFP filter.tif]

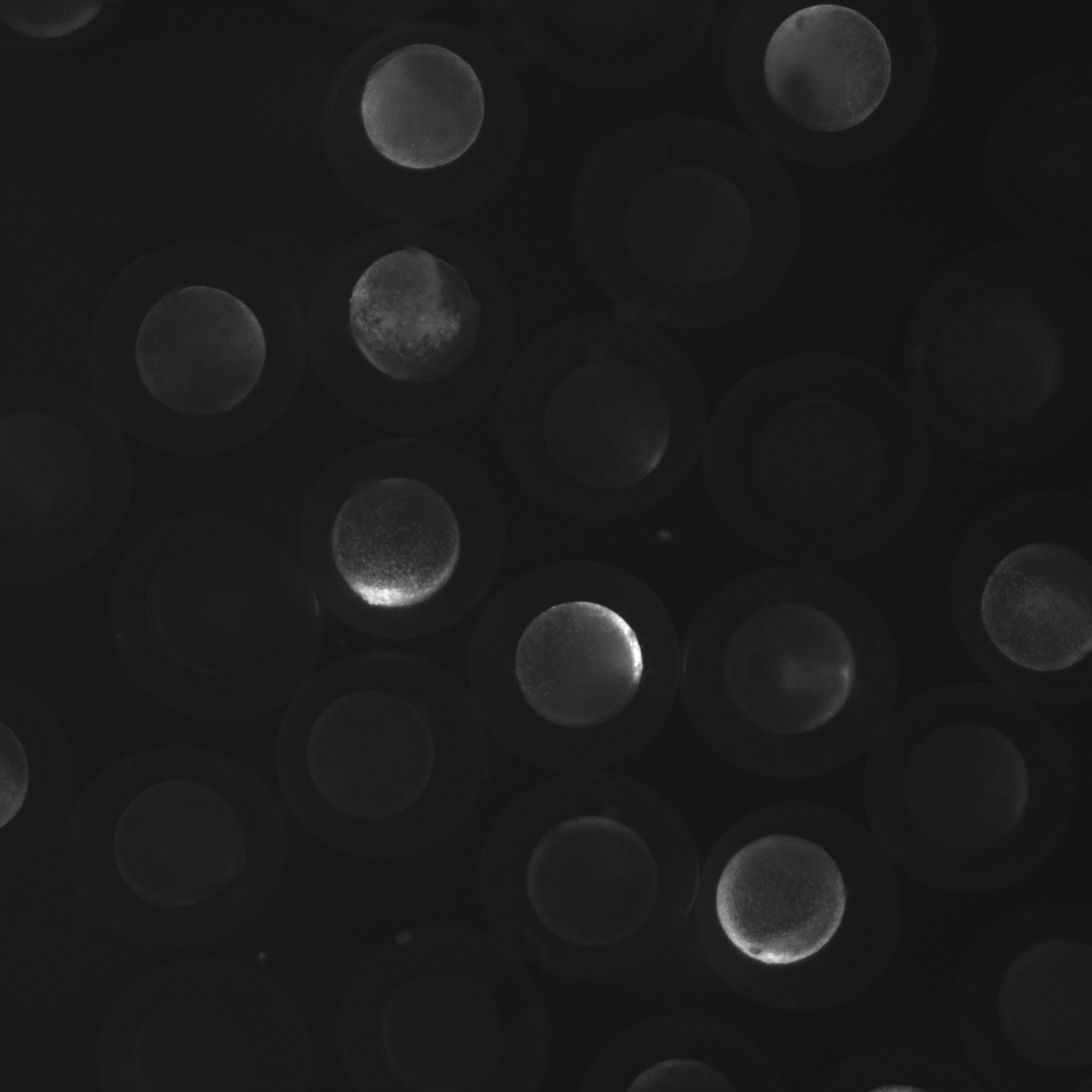

Supplement: Supplementary file 2 — Source data [file 44319_2024_350_MOESM2_ESM.zip › source data/Figure 5/5A/Zebrafish + Potitive Control mimiR_mCherry filter.tif]
