# Supplementary material for: miRNA-target complementarity in cnidarians resembles its counterpart in plants
Source: EMBO Rep. 2025 Jan 2;26(3):836–59. doi: 10.1038/s44319-024-00350-z (PMC11811051; doi:10.1038/s44319-024-00350-z)
Supplement: Supplementary file 3 — Expanded View Figures [file 44319_2024_350_MOESM3_ESM.pdf]

Expanded View Figures

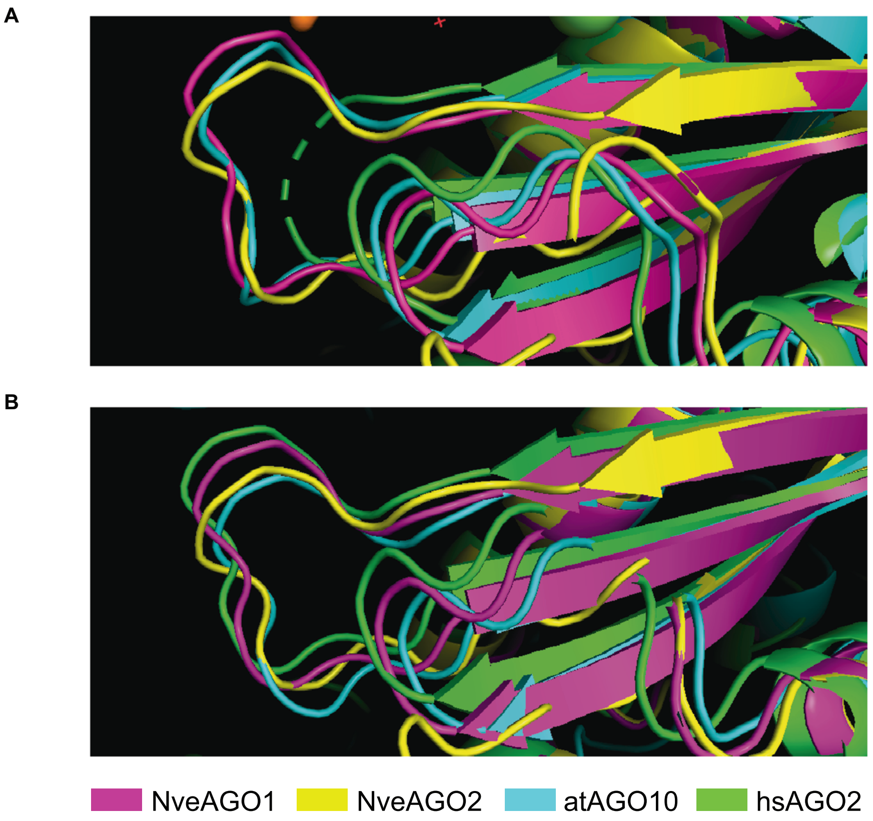

Figure EV1. Structure prediction of *Nematostella* AGOs (NveAGO) shows loop in the PIWI domain.

(**A**) Crystalized structures of atAGO10 and hsAGO2 aligned with Colabfold predicted structures of NveAGO1 and NveAGO2. The PIWI loop appears in atAGO10, NveAGO1 and NveAGO2 but is unstructured in hsAGO2. (**B**) Aligned ColabFold predicted structures of atAGO10, hsAGO2, NveAGO1, and NveAGO2. The PIWI loop appears in all AGOs.

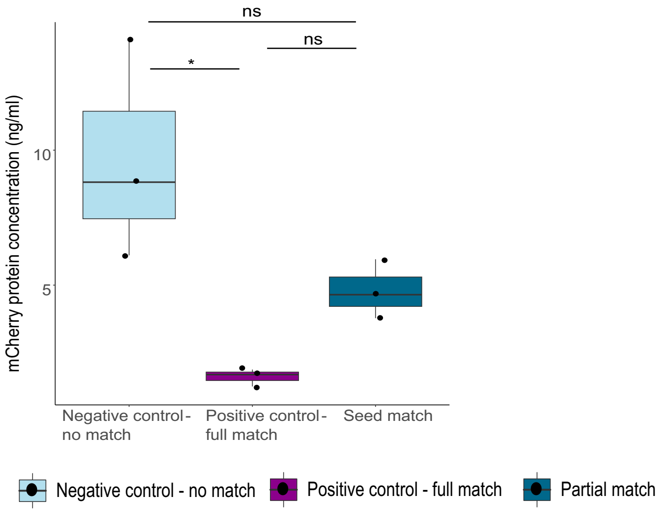

Figure EV2. mCherry mRNA expression in *Hydractinia* is knocked down by nearly-full match mimiR.

mCherry protein concentration measured 24 h after injection with mCherry mRNA combined with different mimiRs to *Hydractinia* zygotes. Measured by ELISA assay. Significance is shown for pairwise comparisons (one-way ANOVA with Tukey's HSD post hoc test, $n = 3$ biological replicates). *P* values are: positive control—partial match 0.1583761, negative control—partial match 0.3124345, negative control—positive control 0.0155945. Data information: box plots show median, the lower and upper bounds correspond to the 25th and 75th percentiles and whiskers extend to maximum and minimum values. Statistically significant difference is represented by: *P value < 0.05, ns not significant. Source data are available online for this figure.

 © The Author(s)

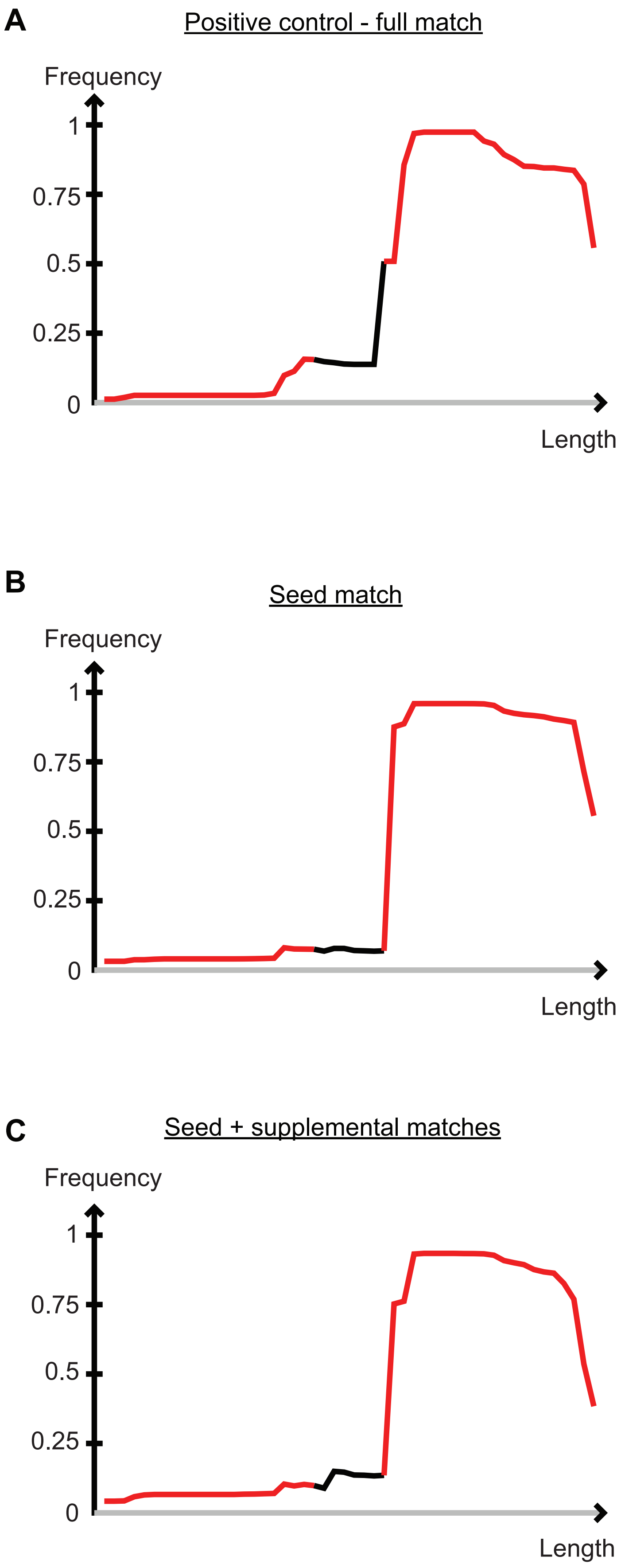
A
Positive control - full match
Frequency
1
0.75
0.5
0.25
0
Length
B
Seed match
Frequency
1
0.75
0.5
0.25
0
Length
C
Seed + supplemental matches
Frequency
1
0.75
0.5
0.25
0
Length

Figure EV3. Processing of mimiRs injected to *Nematostella.*

(**A**) Nearly-full match mimiR (positive control) reads mapped to injected precursor. (**B**) Seed match mimiR reads mapped to injected precursor. (**C**) Seed + supplemental matches mimiR reads mapped to injected precursor.

 © The Author(s)
